# Pan-Cancer Transcriptome and Immune Infiltration Analyses Reveal the Oncogenic Role of Far Upstream Element-Binding Protein 1 (FUBP1)

**DOI:** 10.3389/fmolb.2022.794715

**Published:** 2022-02-22

**Authors:** Huan Wang, Rui Zhang, Erliang Li, Rongbao Yan, Baoan Ma, Qiong Ma

**Affiliations:** Department of Orthopedic Surgery, Orthopedic Oncology Institute, Tangdu Hospital, Fourth Military Medical University, Xi’an, China

**Keywords:** FUBP1, prognosis, protein phosphorylation, DNA methylation, genetic alteration, immune infiltration

## Abstract

Despite increasing evidence to support the relationship between FUBP1 and tumorigenesis in some types of cancers, there have been no analyses from a pan-cancer perspective. Here, we are the first to investigate the putative oncogenic role of FUBP1 in 33 cancer types based on The Cancer Genome Atlas (TCGA) and Gene Expression Omnibus (GEO) databases. Dysregulated *FUBP1* expression was observed in most cancer types, and high *FUBP1* expression suggests poor prognosis in cancers such as ACC, KICH, LIHC, LUAD, LUSC, SARC, CESC, and SKCM. Missense mutation is the most common type of FUBP1 mutation, and R430 in KH_4 is a predominant mutation site. Enhanced phosphorylation of FUBP1 at the S120 site has been observed in clear cell RCC, lung adenocarcinoma, and pediatric brain cancer specimens from African-American and Asian individuals. The expression of *FUBP1* was found to be negatively correlated with the infiltration of CD8^+^ T lymphocytes in GBM, HNSC-HPV- and UCEC but positively correlated with that of tumor-associated fibroblasts in CESC, ESCA, HNSC, LIHC, LUAD, PAAD, and THYM. Furthermore, RNA splicing and spliceosome signaling were predominantly enriched in both GO and KEGG analyses of the functional mechanism of FUBP1. Briefly, this pan-cancer analysis comprehensively revealed the multifaceted characteristics and oncogenic role of FUBP1 in different human cancers.

## Introduction

In view of the complexity of tumorigenesis and development, it is of vital significance to perform pan-cancer analyses of target genes, evaluate the correlation with clinical prognosis and reveal the potential molecular mechanisms (Aran et al., 2015; Cui et al., 2020).

FUBP1 (far upstream element-binding protein 1) is a DNA- and RNA-binding protein contributing to various normal and pathological biological activities and is believed to function as an oncogenic factor in hematologic disorders and solid tumors, acting as either an activator or repressor of transcription (Zheng et al., 2020). It has been reported that knockdown of *FUBP1* in murine models of chronic and acute myeloid leukemia as well as leukemia cells resulted in prolonged survival and decreased cell cycle activity (Hoang et al., 2019). In neuroblastoma, FUBP1 was found to promote the development of tumor cells by targeting HIF-1α and enhancing glycolysis (Jiang et al., 2019). Moreover, the noncoding RNAs LCAT3 and circACTN4 can interact with FUBP1 and thereby activate c-MYC in lung cancer and breast cancer respectively (Qian et al., 2021; Wang et al., 2021b). Our team has focused on the multifunctional FUBP1 protein and has reported on the links between FUBP1 and chemoresistance in human osteosarcoma cells (Wang et al., 2021a). However, no pan-cancer analyses have been conducted to explore the relationship between FUBP1 expression and various cancer types based on clinical data.

We explored TCGA, GTEx, and GEO databases in this study to perform a pan-cancer analysis of FUBP1 to investigate its gene expression level and relationship with pathological staging and prognosis, genetic alteration, DNA methylation, protein phosphorylation, and immune infiltration to uncover the potential molecular mechanism of *FUBP1* in different tissues as well as its significance for clinical prognosis.

## Materials and Methods

### Gene Expression Analysis

The “Diff Exp” module of the website TIMER (http://timer.cistrome.org/) was utilized to explore the expression of *FUBP1* in various types of primary tumors and normal control tissues in the TCGA database. The “Expression DIY-Box Plots” module of the GEPIA2 web server (http://gepia2.cancer-pku.cn/) (Tang et al., 2019) was used to analyze the difference in the expression of *FUBP1* in various tumors when normal tissues were difficult to obtain. The settings were as follows: *p* value cutoff = 0.01, fold change ≥2, and “match TCGA normal and GTEx data.” Log_2_ (TPM+1) was used to represent the expression level of *FUBP1*. Cancer cell line encyclopedia (CCLE) (http://portals. broad institute.org/ccle) (Barretina et al., 2012) was explored to investigate the expression of *FUBP1* in different tumor cell lines. The UALCAN web server (http://ualcan.path.uab.edu/) was applied to analyze the total protein expression level of FUBP1 between the primary cancer tissue and normal tissues using data from the CPTAC (Clinical Proteomic Tumor Analysis Consortium) (Chen et al., 2019). The correlation between the expression of *FUBP1* and the clinical pathological stage in different tumor types was analyzed using the “stage plot” of GEPIA2.

### Survival Prognosis Analysis

The “survival map” and “survival analysis” modules of GEPIA2 were utilized to obtain the OS and DFS maps of *FUBP1* expression in all cancers of TCGA project. The cohorts were divided into high-expression and low-expression groups based on the median group cutoff value (50%–50%). The survival differences between the two groups were analyzed by the log-rank test, and a *p* value less than 0.05 was considered to indicate a significant difference.

### Genetic Alteration Analysis

The cBioPortal web server (http://www.cbioportal.org/) (Cerami et al., 2012; Gao et al., 2013) was used to investigate the genetic alteration of *FUBP1* in different cancers using the “TCGA Pan Cancer Atlas Studies.” “Cancer Type Summary,” “mutations,” and “comparison” modules were used to acquire the data, including alteration frequency, copy number alteration, mutation types and proportion, mutation sites, and the differences in overall survival, progression-free survival, disease-specific survival, and disease-free survival with or without *FUBP1* alteration. The three-dimensional structure of FUBP1 was constructed using the SWISS-MODEL webserver and Swiss-PdbViewer software (https://www.swissmodel.expasy.org, RRID: SCR_013295). Survival curves related to FUBP1 alteration across pan-cancers were generated with the “Comparison/Survival” module of the cBioPortal website.

### DNA Methylation Analysis

The MEXPRESS website (http://mexpress.be/) (Koch et al., 2019) was applied to analyze the DNA methylation of *FUBP1* with various probes in all cancers in TCGA. Each significant result in different cancers was extracted, considering both the correlation coefficient and the *p* value, and a heatmap was generated. The “boxplot, ggpubr, and ggplot2” R package was used to show the DNA methylation status of *FUBP1* in the specific GSE dataset. Wilcoxon test and paired *t*-test were used to calculate the difference.

### Protein Phosphorylation Analysis

CPTAC analysis of the UALCAN portal (RRID: SCR_015827) was explored to analyze the expression level of phospho-FUBP1 between the primary tumor and normal tissues in all cancer types in TCGA. The normalized Z value was used to show the results. The integral domains of FUBP1 with prominent phosphorylation sites were demonstrated using NCBI (https://www.ncbi.nlm.nih.gov/) and IBS (http://ibs.biocuckoo.org/) (Liu et al., 2015).

### Immune Infiltration Analysis

The TIMER2.0 web server was used to investigate the correlation between FUBP1 and the infiltration of immune cells in all cancers in TCGA. The “Immune-Association” module was utilized, and CD8^+^ T cells, fibroblasts, neutrophils, and macrophages were selected for study. Immune cell infiltration was evaluated by the TIMER, EPIC, MCPCOUNTER, CIBERSORT, QUANTISEQ, and XCELL algorithms. The findings were considered reliable when similar results were obtained using at least two algorithms. *p* values were calculated by Spearman’s rank correlation test.

### FUBP1-Related Gene and Pathway Enrichment

The STRING webserver (http://string-db.org) was searched by querying “FUBP1” and “*Homo sapiens.*” The interaction score was set to “medium confidence 0.40,” the network type was set to “full STRING network,” and the maximum number of interactors was set to “50.” The “Similar Gene Detection” module in GEPIA2 was used to acquire the top 100 FUBP1-related genes according to TCGA data. Intersections between the genes in TCGA and STRING were obtained and explored again in TCGA datasets. Pearson correlation analysis of the relationship between *FUBP1* and the intersecting genes was performed using the “correlation analysis” module. Log_2_ (TPM) was used to represent the expression level of each gene. The Metascape web server (http://metascape.org/) (Zhou et al., 2019) was employed for gene ontology analysis. KEGG analysis was performed using the “clusterprofiler” R package.

### Immunohistochemical Staining of Clinical Specimens

Specimens were collected from patients who underwent tumor resection at Tangdu Hospital between January 2017 and December 2020. Studies on the clinical specimens were conducted in accordance with the Declaration of Helsinki, and the protocol was approved by the ethics committee of the Fourth Military Medical University. Paraffin-embedded tumor tissues and healthy control specimens were sliced into 3-μm sections (LEICA, Germany) and heated at 60°C for at least 2 h. Tissues were then deparaffinized and dehydrated using a graded alcohol solution and prepared in accordance with a previously described protocol (Zhang et al., 2021). The anti-FUBP1 (Abcam, Cat. # ab 181111) antibody was used at a dilution of 1:250.

## Results

### Profile of Gene Expression in 33 Human Cancers

The oncogenic role of human FUBP1 (NM_003902.5 or for mRNA or NP_003893.2 for proteins, Supplementary Figure S1A) was investigated, and it was found that the structure of the FUBP1 protein is highly conserved among various species (Supplementary Figure S1B). The phylogenetic tree demonstrated the evolutionary relationship of the protein FUBP1 in different species (Supplementary Figure S2). The expression pattern of FUBP1 in different normal tissues and cells was analyzed using the Human Protein Atlas (HPA), functional annotation of the mammalian genome 5 (FANTOM5), and GTEx database. FUBP1 showed the highest expression in the bone marrow, retina, and ovary and the lowest expression in the olfactory bulb and tongue. Although FUBP1 was expressed in most of the detected tissues and cells, there was low tissue and cell specificity (Supplementary Figures S3A,B). In addition, the expression level of *FUBP1* was explored using the CCLE database, and it was demonstrated that FUBP1 was highly expressed in embryonal cancer, neuroblastoma and leukemia compared with other solid tumors (Supplementary Figure S3C).

**FIGURE 1:**
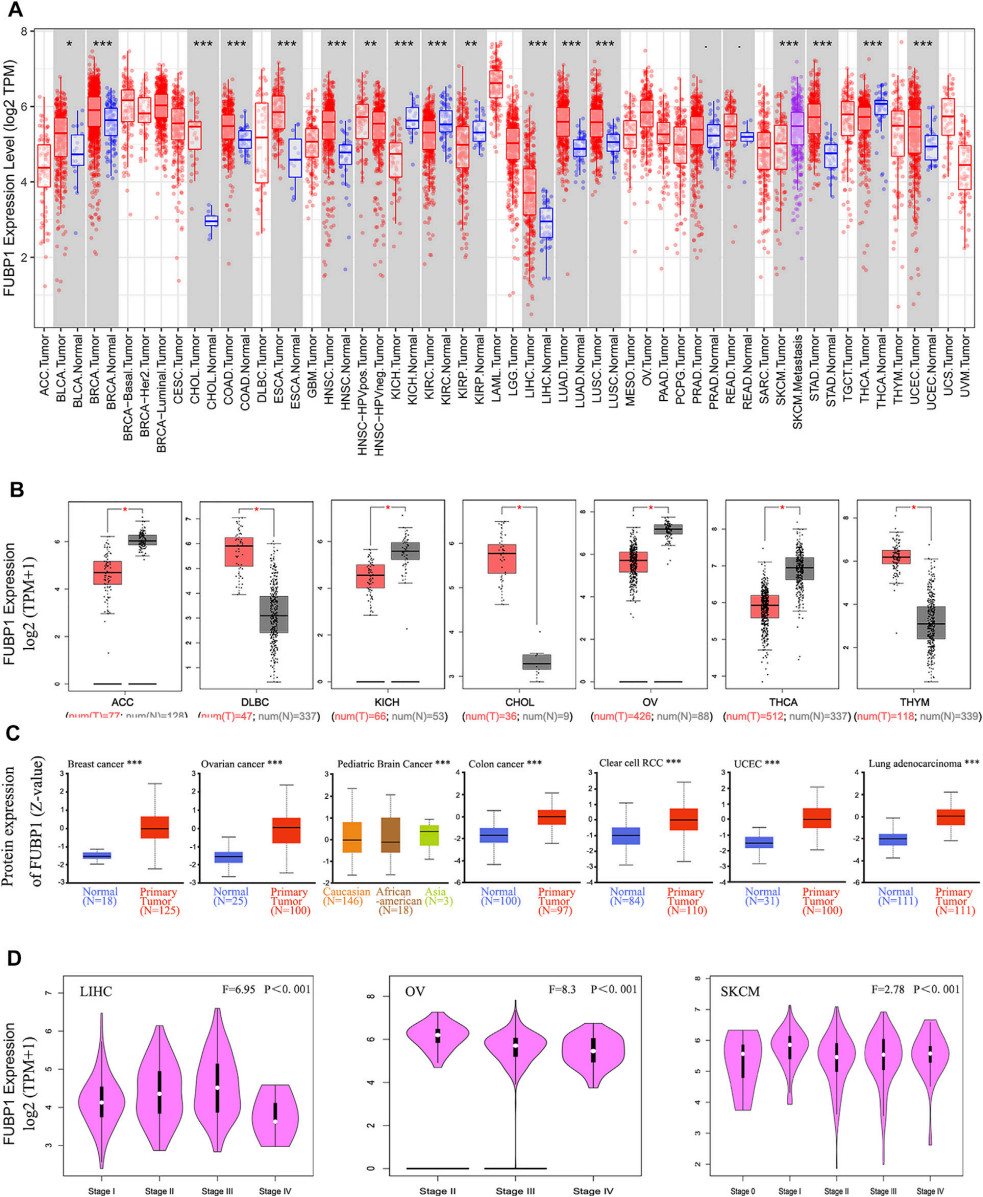
Expression of FUBP1 according to cancer type and clinical pathological stage in TCGA datasets. **(A)** The specific expression level of *FUBP1* in different cancer types and corresponding normal tissues in TCGA database was analyzed using TIMER2. **(B)** GTEx datasets were used to analyze the expression of *FUBP1* in ACC, DLBC, KICH, CHOL, OV, THCA, and THYM. Log_2_ (TPM+1) was applied to represent the expression level. **(C)** The total protein expression level of FUBP1 was analyzed between the primary tumor and the corresponding normal tissues with the CPTAC data. **(D)** The expression of FUBP1 in different clinical stages of LIHC, OV, and SKCM was quantified with log_2_ (TPM+1) and visualized by GEPIA2. *p < 0.05, **p < 0.01, ***p < 0.001.

Next, the expression of FUBP1 in various cancers in TCGA was analyzed using TIMER2. Significantly higher FUBP1 expression was observed in the clinical tissues of BLCA (bladder urothelial carcinoma), BRCA (breast invasive carcinoma), CHOL (cholangiocarcinoma), COAD (colon adenocarcinoma), ESCA (esophageal carcinoma), HNSC (head and neck squamous cell carcinoma), LIHC (liver hepatocellular carcinoma), LUAD (lung adenocarcinoma), LUSC (lung squamous cell carcinoma), STAD (stomach adenocarcinoma), and UCEC (uterine corpus endometrial carcinoma) compared with corresponding normal tissues (Figure 1A, *p* < 0.05).

With the TCGA and GTEx data, the expression of *FUBP1* in tumor tissues and adjacent normal tissues was further analyzed. There was a remarkably higher expression of *FUBP1* mRNA in DLBC (lymphoid neoplasm diffuse large B-cell lymphoma), CHOL, and THYM (thymoma) (*p* < 0.05) and lower expression in ACC (adrenocortical carcinoma), KICH (kidney chromophobe), OV (ovarian serous cystadenocarcinoma), and THCA (thyroid carcinoma) (Figure 1B). There were no significant differences in other tumors, such as ESCA, GBM (glioblastoma multiforme), or HNSC, as shown in Supplementary Figure S4A. The pooled analysis of different reports in the Oncomine database also revealed higher *FUBP1* expression in liposarcoma, salivary gland adenoid cystic carcinoma, T-cell acute lymphoblastic leukemia, rectal mucinous adenocarcinoma, and ovarian serous cystadenocarcinoma than in normal controls (Supplementary Figure S5, *p* < 0.001).

At the protein level, FUBP1 was highly expressed in the primary tumors of breast cancer, ovarian cancer, pediatric brain cancer, colon cancer, clear cell renal cell carcinoma, uterine corpus endometrial carcinoma, and lung adenocarcinoma compared with normal tissue counterparts using the CPTAC dataset (Figure 1C, *p* < 0.001).

The correlation between *FUBP1* and the clinical pathological stage was analyzed using GEPIA2, and the results (medians and *p* values) showed that the expression of *FUBP1* was positively correlated with the cancer stage from stage I to stage III in LIHC and from stage 0 to stage I and from stage II to III in SKCM (skin cutaneous melanoma), while it was negatively correlated with the clinical stage in OV (*p* < 0.001) (Figure 1D) but not in other cancer types (Supplementary Figures S4B–E).

### Survival-Associated Expression of *FUBP1* in 33 Cancer Types

**FIGURE 2:**
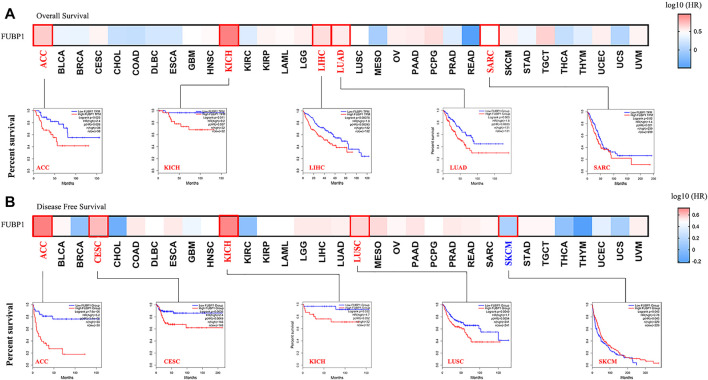
Correlation between FUBP1 expression and the clinical survival prognosis of different cancers based on TCGA datasets. GEPIA2 was utilized to obtain **(A)** overall survival and **(B)** disease-free survival data regarding the expression level of FUBP1 in different tumor types in TCGA. Kaplan–Meier curves were generated, and *p* values less than 0.05 were considered significant.

Clinical tissue specimens were divided into high-expression and low-expression groups, and both OS (overall survival) and DFS (disease-free survival) were investigated in different cancer types using TCGA and GEO datasets to reveal the correlation between *FUPB1* expression and cancer prognosis. OS analysis data demonstrated that high *FUBP1* expression was significantly correlated with poor prognosis in ACC, KICH, LUAD, SARC (sarcoma) (*p* < 0.05), and LIHC (*p* < 0.001) (Figure 2A). Regarding DFS, high *FUBP1* expression conferred a short survival time in ACC (*p* < 0.001), CESC (cervical squamous cell carcinoma and endocervical adenocarcinoma), LUSC (*p* < 0.01), and KICH (*p* < 0.05) (Figure 2B). In contrast, low *FUBP1* expression was related to poor prognosis for MESO (mesothelioma), and READ (rectum adenocarcinoma) patients, according to overall survival, as well as SKCM patients (*p* < 0.05), according to DFS.

Survival analyses were also performed using the Kaplan–Meier plotter tool. High expression of *FUBP1* correlated with poor OS and RFS (relapse-free survival) in cervical squamous cell carcinoma, esophageal adenocarcinoma, kidney renal papillary cell carcinoma, liver hepatocellular carcinoma, lung adenocarcinoma, pancreatic ductal adenocarcinoma, sarcoma, pheochromocytoma and paraganglioma (*p* < 0.05). A relatively good prognosis was observed for breast cancer, esophageal squamous cell carcinoma, kidney renal clear cell carcinoma, rectum adenocarcinoma, and thymoma (*p* value of OS < 0.05). Nonsignificant differences in survival status between the patients with high and low *FUBP1* expression were observed in cancer types such as bladder carcinoma, head-neck squamous cell carcinoma, lung squamous cell carcinoma, ovarian cancer, stomach adenocarcinoma, testicular germ cell tumor, thyroid carcinoma, and uterine corpus endometrial carcinoma (*p* > 0.05) (Supplementary Figure S6). Subgroup analyses of different cancers were also conducted (Supplementary Tables S1–S4), and the results further verified that although high *FUBP1* expression correlates with poor prognosis in most cancer types in TCGA, differences are observed depending on the cancer type and even the subtype.

### Genetic Alteration of *FUBP1* in Different Cancer Types

**FIGURE 3:**
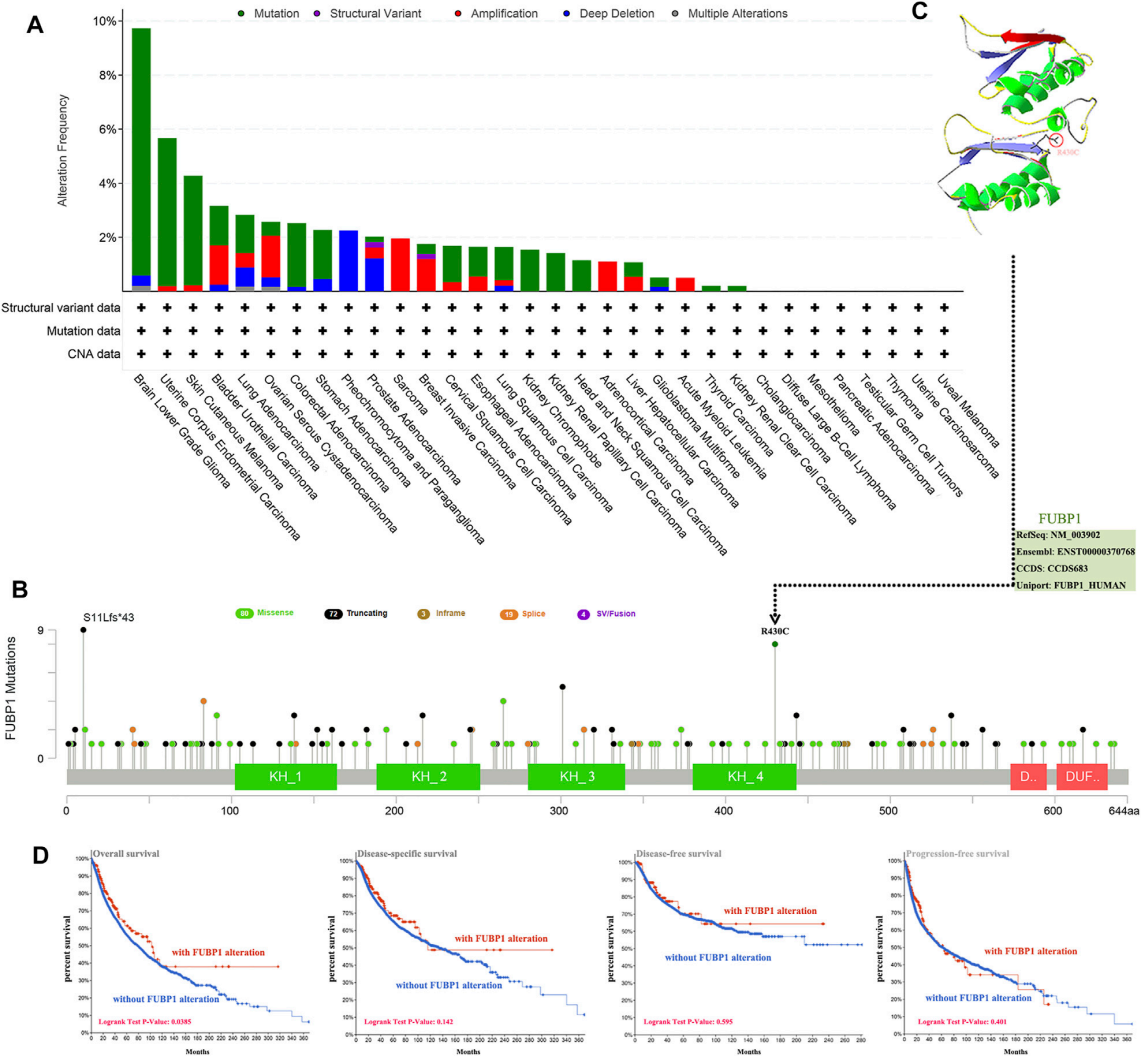
Mutation features of FUBP1 in different cancer types in TCGA. **(A)** cBioPortal was used to analyze the genetic alterations of FUBP1. **(B)** The alteration frequency and type as well as the specific sites with the highest alteration frequency (R430C) are displayed. **(C)** The 3-D structure of the FUBP1 protein was acquired using the SWISS-MODEL tool and Swiss-PdbViewer software. **(D)** Correlations between FUBP1 mutation and overall survival, disease-specific survival, disease-free survival and progression-free survival in pan-cancer were analyzed using the cBioPortal tool.

The genetic alteration of *FUBP1* in different cancers was investigated according to the clinical datasets in TCGA, the highest alteration frequency for FUBP1 appeared in patients with low-grade glioma, showing an alteration frequency of nearly 10%, while no alteration in FUBP1 was observed in patients with uveal melanoma, uterine carcinosarcoma, thymoma, testicular germ cell tumors, pancreatic adenocarcinoma, mesothelioma, diffuse large B-cell lymphoma, or cholangiocarcinoma. It was also found that all sarcoma and acute myeloid leukemia patients with genetic alterations exhibited amplification-type copy number aberrations (approximately 2.5% and 1%, respectively), and all pheochromocytoma and paraganglioma patients showed deep deletion-type genetic alterations (approximately 2.7%) (Figure 3A). Figure 3B presents the alteration types and specific sites in *FUBP1*. Missense mutation and truncation are the two primary genetic alteration types, in which R430C alteration in the KH_4 domain and truncation at S11Lfs*43 are predominant. The R430C site is depicted in a three-dimensional structure of FUBP1 in Figure 3C. We also found that sv/fusion and in-frame mutations were the least common alterations in *FUBP1*. Then, the association between genetic alterations in *FUBP1* and clinical prognosis was explored, and the results indicated that cancer patients with *FUBP1* alteration had better overall survival than patients without *FUBP1* alteration (*p* = 0.0385), but no significant differences in disease-specific and disease-free survival or progression-free survival were found (Figure 3D).

Moreover, both the correlation between the expression of *FUBP1* and tumor mutational burden (TMB) and microsatellite instability (MSI) were investigated across all cancers in TCGA. Significant positive correlations between *FUBP1* expression and TMB were found in LUAD (*p* = 5.9e^−06^), LAML (acute myeloid leukemia, *p* = 0.016), SKCM (*p* = 0.0023), STAD (p = 4e^−10^), and COAD (*p* = 0.029), while significant negative correlations were observed in BRCA (*p* = 0.0017), THCA (*p* = 4.6e-07), and KIRC (kidney renal clear cell carcinoma) (*p* = 0.006) (Supplementary Figure S7). Regarding MSI, a positive correlation was found in LUSC, UCEC, READ, STAD, and COAD (*p* < 0.05), and a negative correlation was found in DLBC and HNSC (*p* < 0.05) (Supplementary Figure S8). These findings should be investigated further in different cancer types.

### DNA Methylation of *FUBP1* in Cancer

**FIGURE 4:**
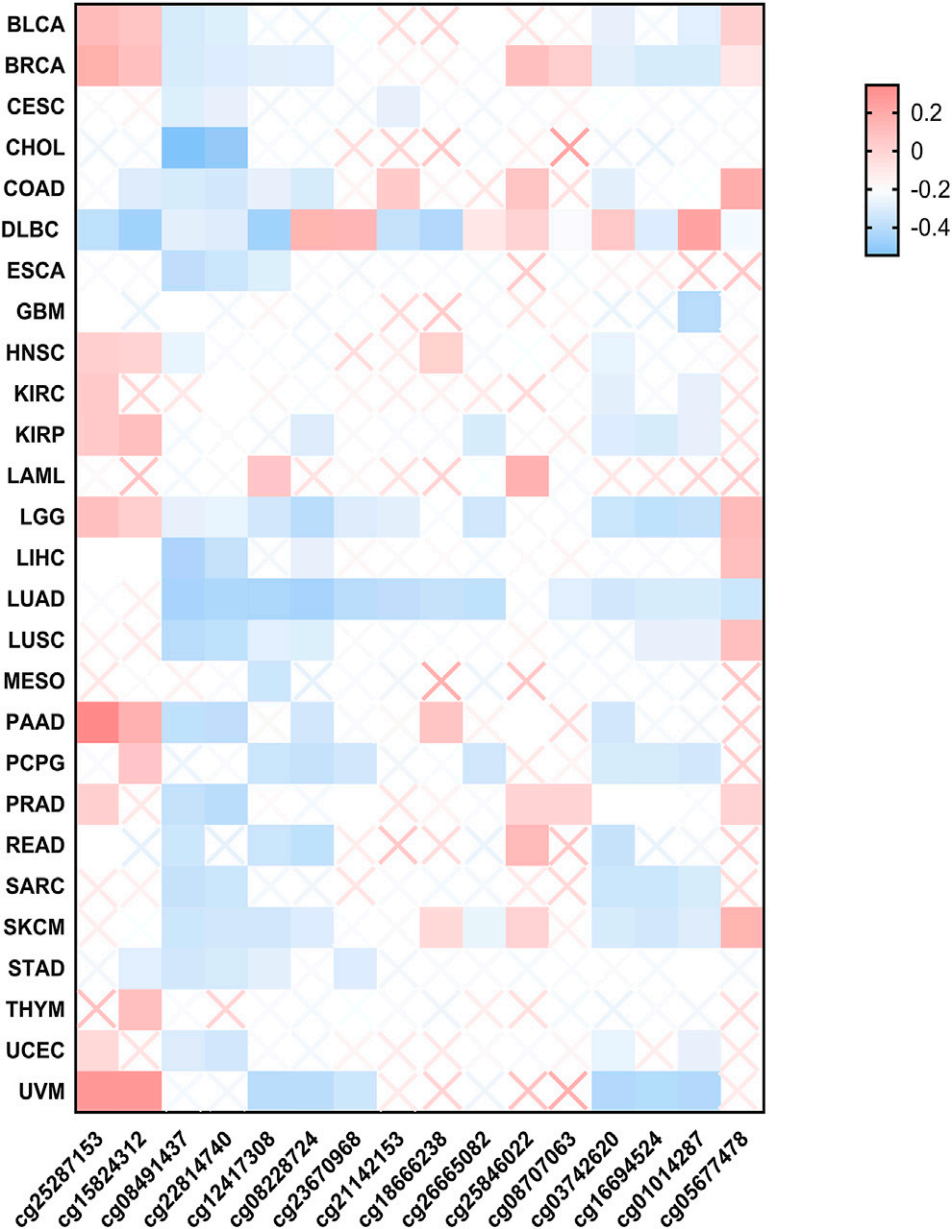
The methylation status of FUBP1 in different cancer types. Multiple probes were used to explore the methylation of FUBP1 *via* MEXPRESS, and the corresponding heatmap data in different cancers were displayed using GraphPad software.

The association between DNA methylation of *FUBP1* and the clinicopathological characteristics of different cancers was determined using MEXPRESS. We found that the methylation level of *FUBP1* was high in BLCA, BRCA, PAAD (pancreatic adenocarcinoma), KIRP (kidney renal papillary cell carcinoma), and UVM (uveal melanoma) with the probes cg25287153 and cg15824312 and was high in COAD, LGG (brain lower grade glioma), LIHC, LUSC, and STAD with the probe cg05677478. In contrast, low methylation levels of *FUBP1* were observed in CHOL, LIHC, LUAD, and UVM. In patients with CESC, CHOL, ESCA, GBM, LUAD, MESO, SARC, and STAD, either low-level methylation or no methylation of *FUBP1* was observed, indicating the high expression of *FUBP1* in the abovementioned cancer types (Figure 4). With respect to the COAD cases, we observed a significant negative correlation between DNA methylation and *FUBP1* expression with most probes. Three probes (cg15824312, cg08491437, and cg22814740) that detected relatively high levels of DNA methylation showed a negative correlation between the methylation level and the expression of *FUBP1* (*p* < 0.01, *p* < 0.001, and *p* < 0.001, respectively) (Supplementary Figure S9). The methylation level of *FUBP1* in COAD tissues and adjacent normal tissues in the GSE131013 dataset was analyzed (Diez-Villanueva et al., 2020; Diez-Villanueva et al., 2021), and a nonsignificant difference was observed based on the normalization of chip data (Supplementary Figures S10A,B, *p* = 0.06). Moreover, we found reduced *FUBP1* methylation in tumor tissues with multiple probes, such as cg22814740 (*p* = 5.28e-05) and cg08491437 (*p* = 1.4e-06) (Supplementary Figure S10C).

### Phosphorylation of FUBP1 in Cancer

**FIGURE 5:**
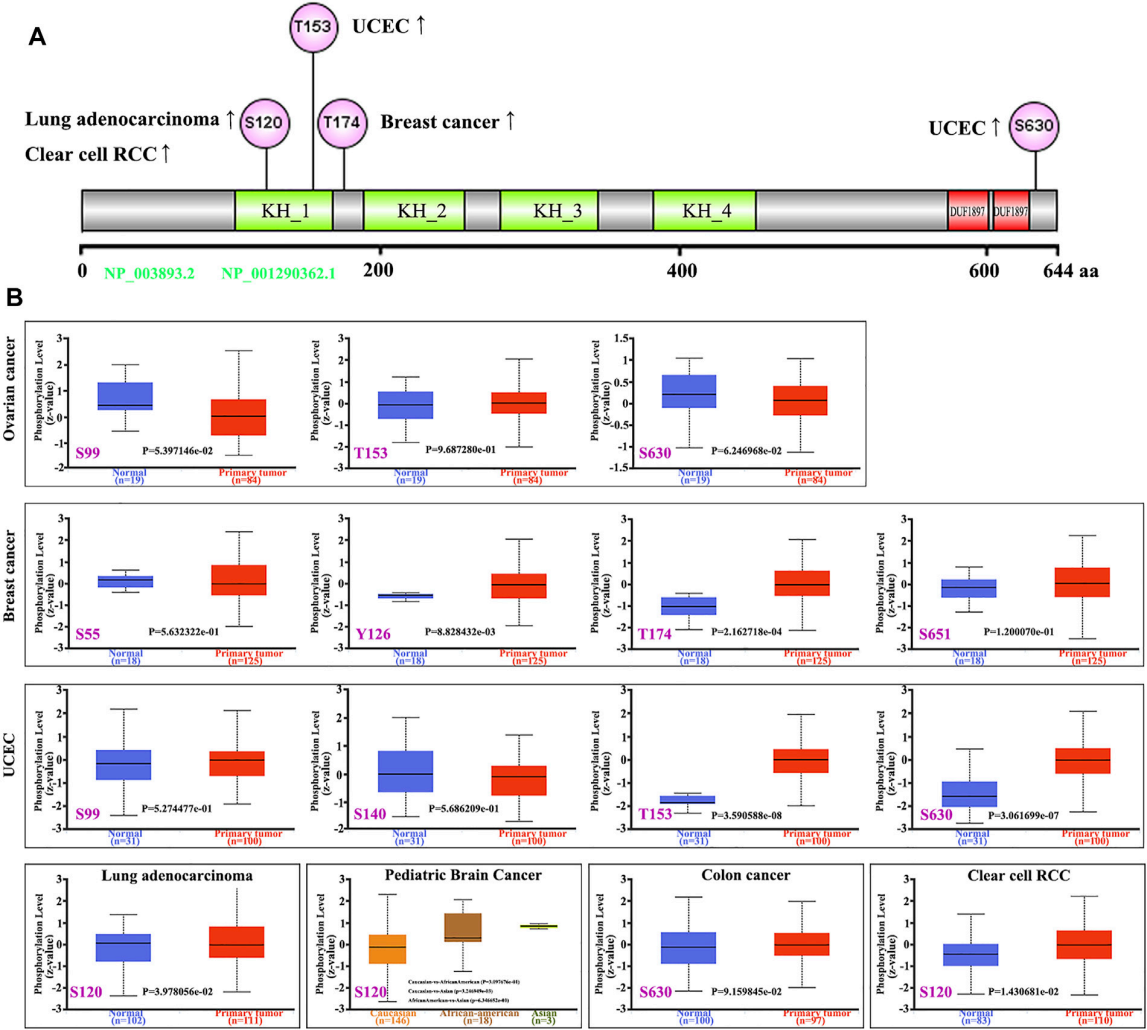
Phosphorylation analysis of the FUBP1 protein in different cancer types. **(A)** The prominent phosphorylation sites are displayed in the structural schematic diagram of the FUBP1 protein. **(B)** The expression level of phosphorylated FUBP1 between primary tumor tissues and corresponding normal tissues was analyzed using CPTAC analysis in UALCAN, and box plots for ovarian cancer, breast cancer, UCEC, lung adenocarcinoma, pediatric brain cancer, colon cancer, and clear cell RCC are displayed.

The phosphorylation level of FUBP1 was analyzed between the primary tumors and their normal counterparts using the CPTAC datasets. The specific phosphorylation sites and significant differences in different cancers are shown in Figure 5. The S120 locus within the KH_1 domain of FUBP1 demonstrated a high phosphorylation level in primary tissues of lung adenocarcinoma (*p* = 0.03978) and clear cell renal carcinoma (*p* = 0.01431) compared with control tissues and in pediatric brain cancer in Asian children compared with Caucasian children (*p* = 0.003247). Elevated phosphorylation levels at S630 were observed in primary UCEC, colon cancer and ovarian cancer tumors. However, the phosphorylation of FUBP1 at this site only showed a significant difference in UCEC (*p* = 3.061e-07) (Figures 5A,B). The PhosphoNET database was utilized to analyze the CPTAC-identified phosphorylation of FUBP1, and the results revealed that the phosphorylation of FUBP1 at S630 was experimentally supported in HeLa cells (Supplementary Table S5). Further exploration of the potential function of S120 and S630 phosphorylation during the process of tumor development in different cancer cell lines is needed in the future.

### Correlation Between FUBP1 and Immune Infiltration in Cancer

**FIGURE 6:**
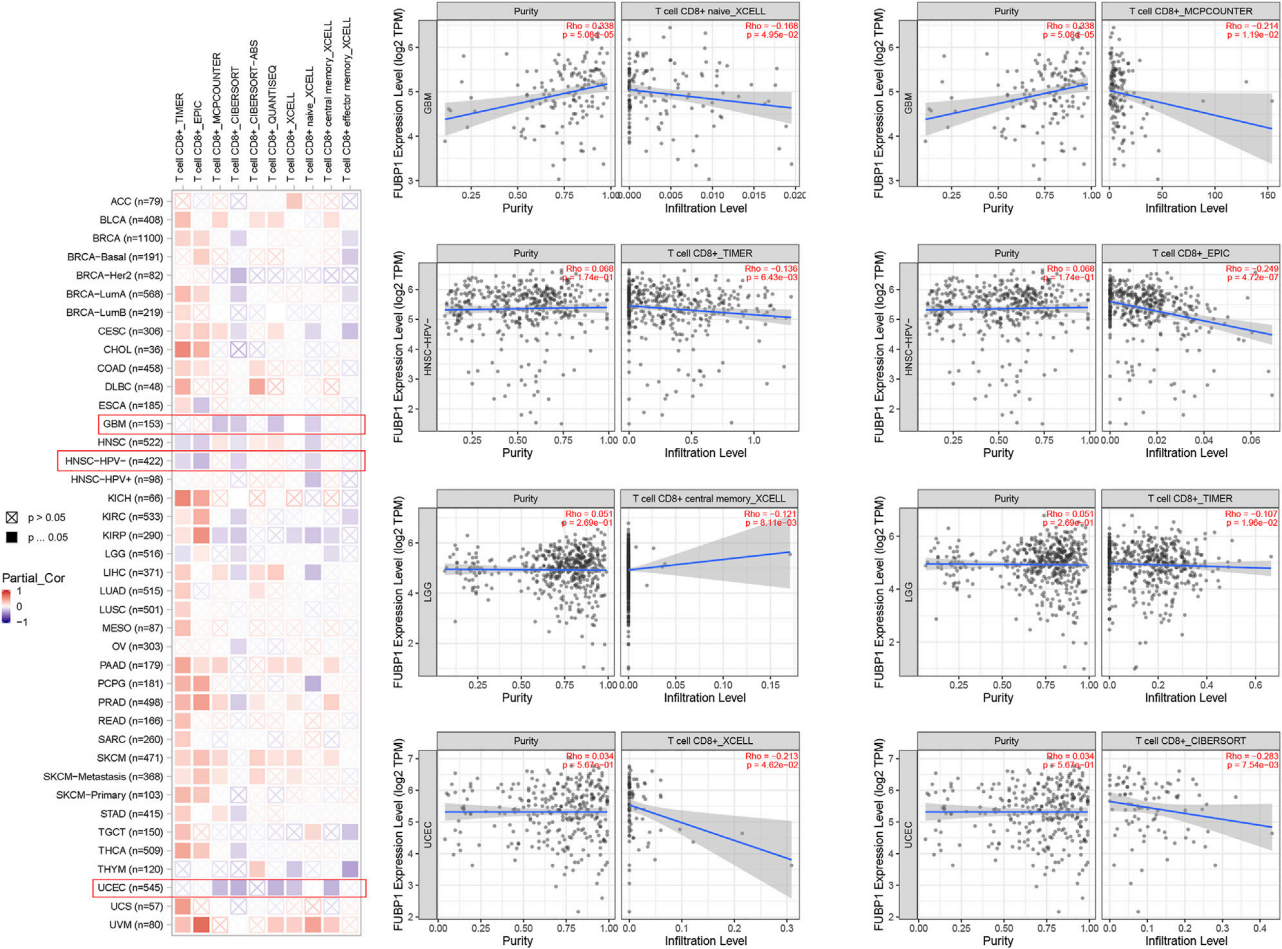
Correlation analysis between FUBP1 expression and the immune infiltration of CD8^+^ T cells. Multiple algorithms were applied to explore the correlation between the expression of *FUBP1* and the infiltration level of CD8^+^ T cells in different cancer types in TCGA.

**FIGURE 7:**
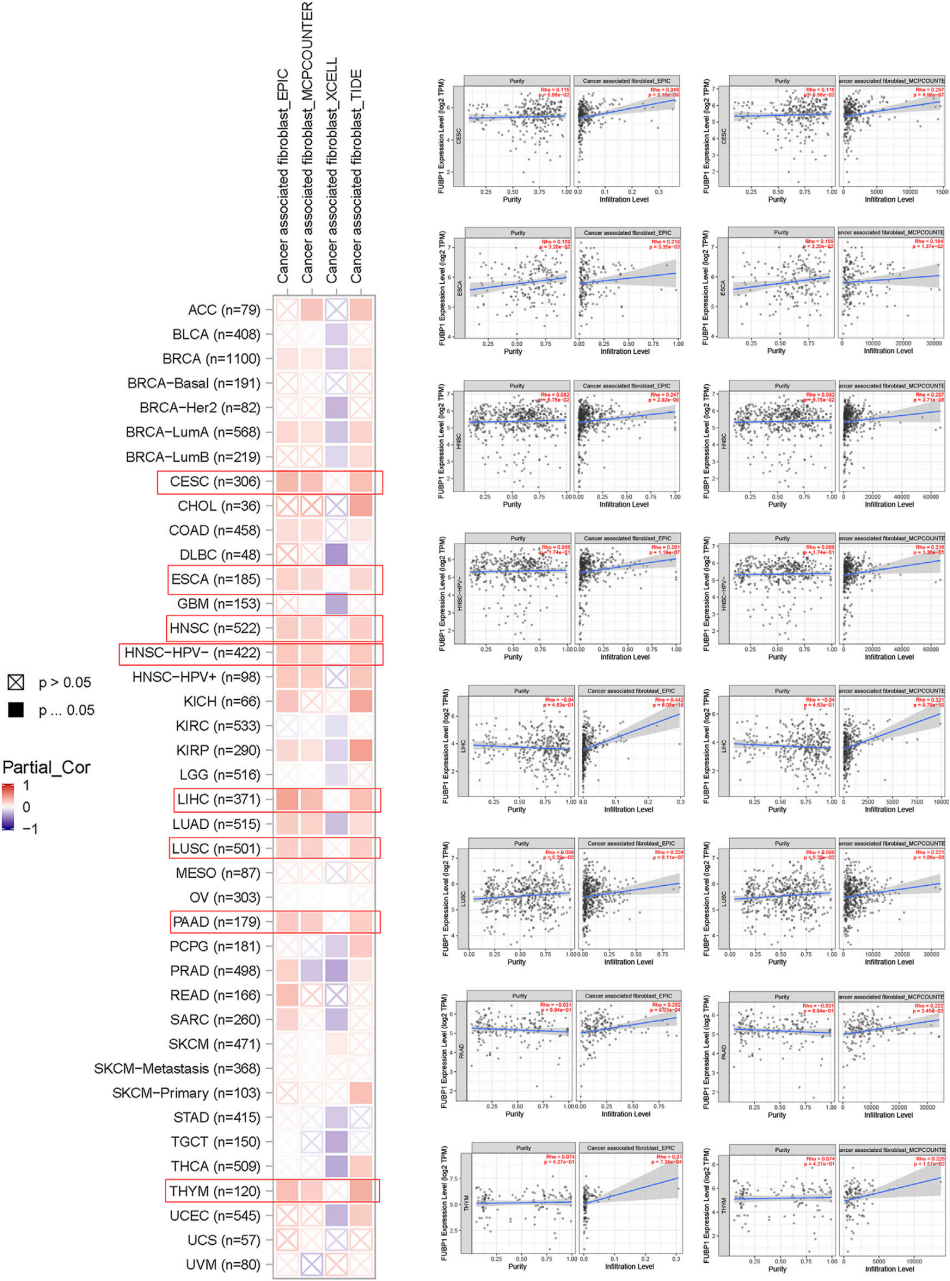
Correlation analysis between FUBP1 expression and the immune infiltration of cancer-associated fibroblasts. Multiple algorithms were applied to explore the correlation between the expression of *FUBP1* and the infiltration level of cancer-associated fibroblasts in different cancer types in TCGA.

Immune cells, which include innate immune cells and adaptive immune cells, are important members of the tumor microenvironment that affect the progression and prognosis of various cancers. CD8^+^ T cells are the most functional T cells in almost all cancers and are recognized as an efficient target for immunotherapy (Farhood et al., 2019). The relationship between FBUP1 expression and immune cells was investigated in different cancers using the TIMER (Tumor Immune Estimation Resource), EPIC, MCPCOUNTER, CIBERSORT, QUANTISEQ, and XCELL algorithms. There was a negative correlation between the infiltration level of CD8^+^ T cells and FUBP1 expression in GBM (*p* = 4.95e^−02^ with XCELL and *p* = 1.19e^−02^ with MCPCOUNTER), HNSC-HPV- (*p* = 6.43e-03 with TIMER and *p* = 4.72e-07 with EPIC), and UCEC (*p* = 4.62e^−02^ with XCELL and *p* = 7.54e-03 with CIBERSORT), but either a negative or positive correlation was observed in LGG with different algorithms (Figure 6). In addition, a significant positive correlation was observed between FUBP1 expression and the infiltration of cancer-associated fibroblasts in CESC, ESCA, HNSC, LIHC, LUSC, PAAD, and THYM with at least three different algorithms (Figure 7). Regarding macrophages, a positive correlation was found for ACC, BLCA, BRCA, COAD, HNSC, MESO, PCPG (pheochromomocytoma and paraganglioma), and UVM, while a negative correlation was found in CHOL, KIRP, UCEC, and UCS (uterine carcinosarcoma) (Supplementary Figures S11, S12)**.**

### FUBP1-Related Partners in Cancer

**FIGURE 8:**
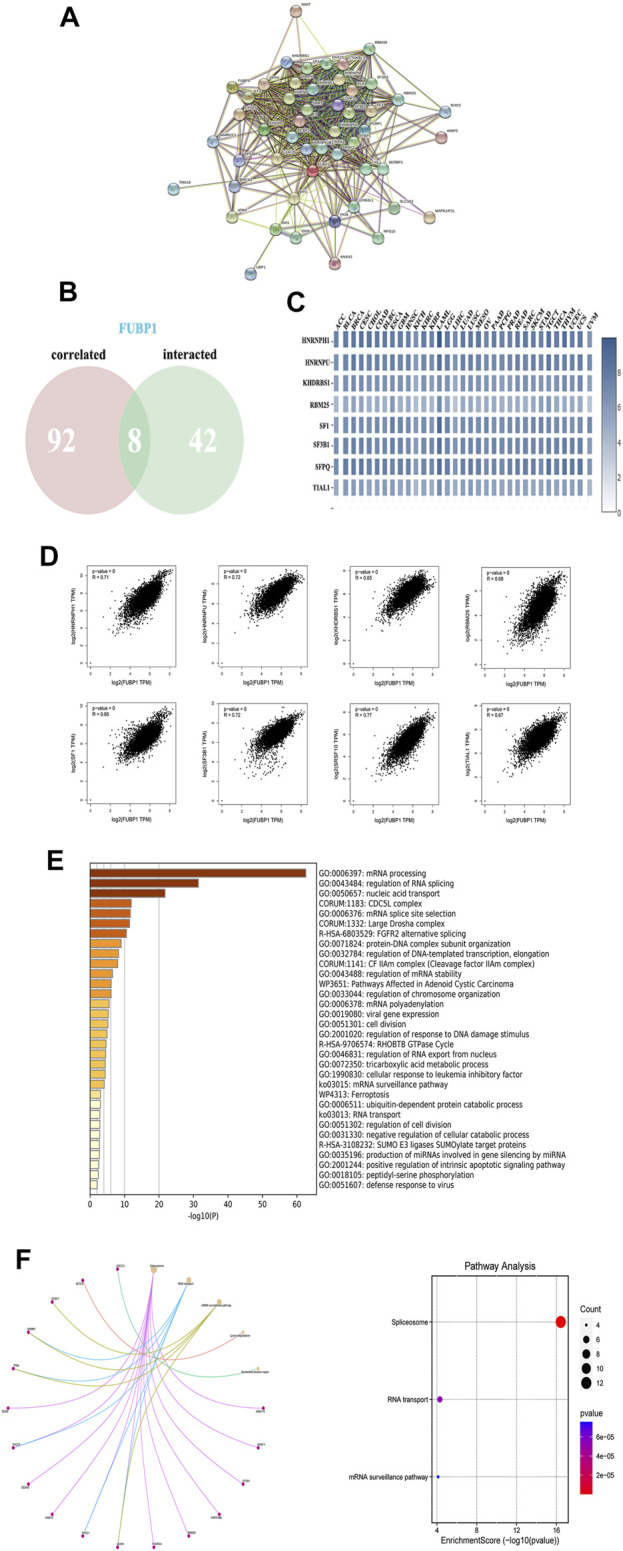
The enrichment of FUBP1-related genes and pathways. **(A)** The PPI network was constructed using the STRING database, and the top 50 proteins are displayed. **(B)** An intersection of eight proteins was obtained from the 100 FUBP1-correlated proteins via GEPIA2 and 50 FUBP1-binding proteins *via* STRING. **(C)** The expression of the eight genes in each cancer type is displayed in a heatmap. **(D)** The potential correlation between FUBP1 and the eight intersecting genes was explored using the Metascape tool. Log_2_TPM was used to represent the expression levels of genes. **(E)** The biological process data in GO analysis were analyzed using Metascape. **(F)** Significantly enriched signaling pathways based on the intersection of FUBP1-binding and interacting genes are shown.

To further explore the molecular mechanism of *FUBP1* in cancer progression, protein-protein interactions (PPIs) were analyzed using STRING (http://string-db.org/). As illustrated, 50 FUBP1-binding proteins were obtained that were either experimentally determined or supported by text mining. The interaction network of these proteins is shown in Figure 8A. In addition, the top 100 genes that correlated with *FUBP1* expression in the TCGA database were collected using GEPIA2. Intersections were taken, and eight partners of *FUBP1* were identified (Figure 8B). As illustrated, the expression of *FUBP1* was positively correlated with that of all eight genes in all 33 cancer types (*p* < 0.001). The genes were as follows: HNRNPH1 (R = 0.71), HNRNPU (R = 0.72), KHDRBS1 (R = 0.65), RBM25 (R = 0.68), SF1 (R = 0.68), SF3B1 (R = 0.72), SFPQ (R = 0.77), and TIAL1 (R = 0.67) (Figures 8C,D). Gene ontology analysis was performed, and mRNA processing, regulation of RNA splicing, and mRNA splice site selection were among the top five enriched GO terms (Figure 8E, Supplementary Figure S13). The KEGG analysis suggested that the oncogenic role of *FUBP1* in different cancers might involve the “spliceosome” (*p* = 9.2e^−25^, 22 genes included) and “mRNA surveillance” (*p* = 3.1e^−3^, 5 genes included) pathways (Figure 8F, Supplementary Figures S14, S15).

### Immunohistochemical Staining of FUBP1 in Clinically Resected Specimens

**FIGURE 9:**
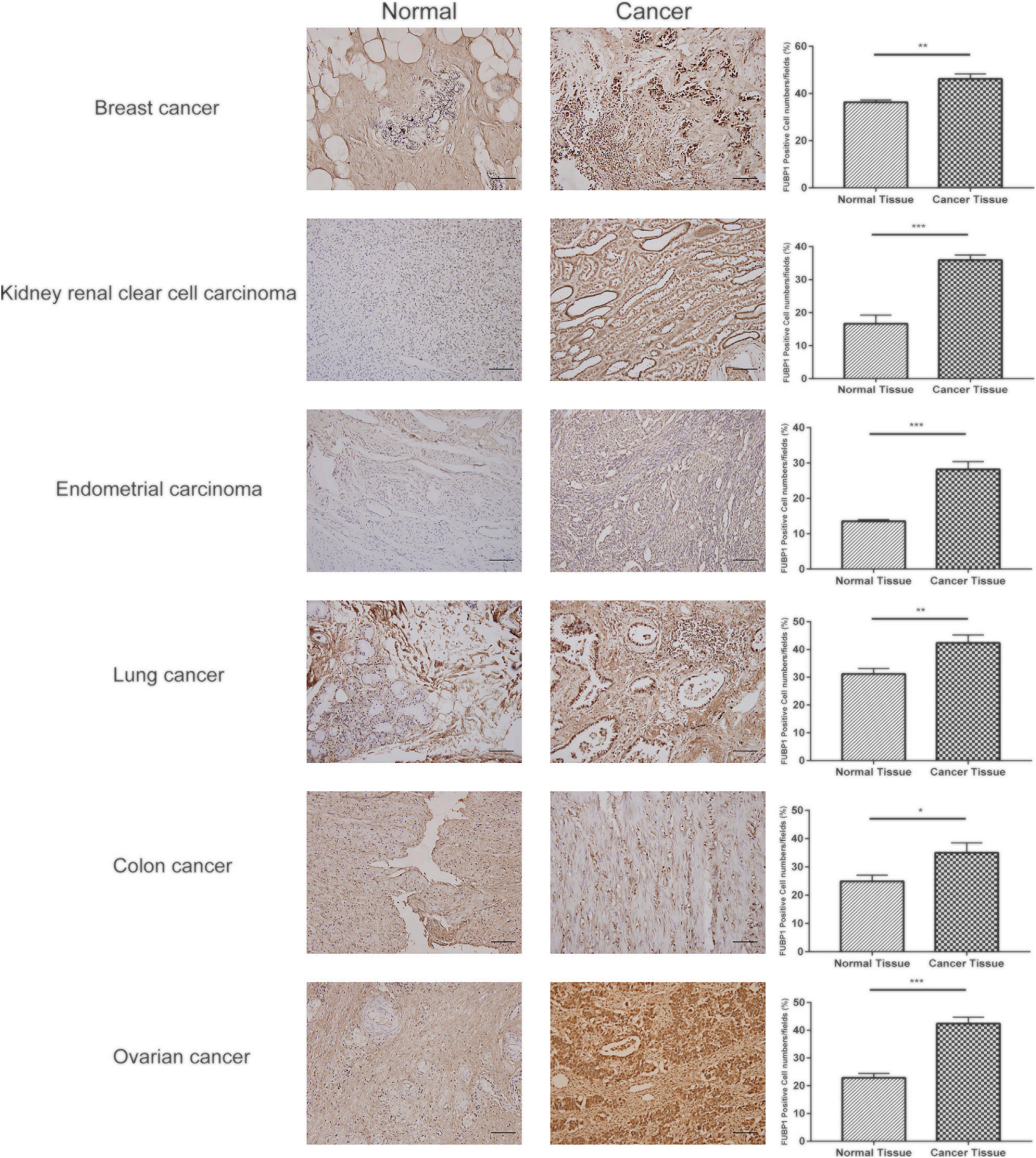
Expression of FUBP1 showed differences between six types of tumor tissues and their normal counterparts. Representative immunohistochemistry staining of clinical specimens; scale bar = 100 μm (magnification, ×200). *p < 0.05, **p < 0.01, ***p < 0.001.

Immunohistochemical staining was used to detect the expression level of FUBP1 in breast cancer, ovarian cancer, colon cancer, clear cell RCC, UCEC, lung adenocarcinoma and normal control tissues. IHC analysis revealed nuclear FUBP1 staining in both tumor and normal tissues. However, the expression of FUBP1 was much stronger in the tumor tissues than in the normal control tissues, which is consistent with the in silico study (Figure 9).

## Discussion

FUBP1 has been reported to be a DNA- and RNA-binding protein involved in the processes of transcription, translation, and alternative splicing (Debaize et al., 2018; Debaize and Troadec, 2019; Elman et al., 2019; Kang et al., 2020). The phylogenetic tree analysis conducted in our study demonstrated the highly conserved nature of this protein across different species, suggesting the shared expression of FUBP1 among common ancestors of humans and animals and indicating that there may be a similar mechanism in various physiological and pathological processes. The existence and function of FUBP1 in cancer have been reported for neuroblastoma, myeloid leukemia, tongue squamous cell carcinoma, glioma, etc. (Cancer Genome Atlas Research et al., 2015; Hoang et al., 2019; Jiang et al., 2019; Chen et al., 2020). However, different molecular mechanisms and binding proteins of FUBP1 were reported for different cancers in the above publications, and there have been no pan-cancer analyses of FUBP1 from an overall perspective. This study was the first multifaceted investigation examining the *FUBP1* gene and protein in all cancers found in TCGA, including the mRNA expression level, survival correlation, mutation, phosphorylated protein, surrounding immune environment, enriched partners and related signaling pathways.

The expression levels of *FUBP1* were significantly higher in eleven clinical tumors than in adjacent normal tissues according to TCGA datasets. However, the survival prognosis showed distinct results. For example, *FUBP1* is positively correlated with overall survival in patients with sarcoma. However, no significant difference was observed in the expression of *FUBP1* between primary tumor tissues and normal controls. One of the possible reasons may be the difficulty in obtaining control tissues for some types of sarcoma and therefore the lack of precise data. In our previous study, we found that a high level of FUBP1 could confer lobaplatin resistance in osteosarcoma cell lines, which resulted in poor outcomes (Wang et al., 2021a). We also compared the expression of the FUBP1 protein in platinum-resistant and platinum-sensitive specimens, and a significant difference was observed.

In addition, the expression of *FUBP1* at the transcriptional level was not always consistent with that at the translational level, as observed in reproductive system tumors (OVs). According to the TCGA and GTEx datasets, the expression of *FUBP1* in ovarian cancer tissue was lower than that in normal tissues. However, the CPTAC data showed the opposite result at the protein level, with a significant difference (*p* < 0.001) in protein phosphorylation at S99, S630, and T153. Proteins are the functional units of biological activity, and phosphorylation is the most pivotal posttranslational modification in the process of protein activation. It is speculated that some posttranscriptional changes occur during the process of tumorigenesis in OV. For liver cancer, TCGA dataset revealed significantly high *FUBP1* expression in LIHC, while no difference was found at the protein level based on the CPTAC data. However, the expression of FUBP1 is closely correlated with the clinical stage, especially from stage I to III in LIHC. FUBP1 increased from a moderate to significantly high level as LIHC progressed (*p* < 0.001), indicating that FUBP1 could be a potential marker for the diagnosis of patients with different disease stages so that corresponding therapeutic strategies could be applied accordingly. Moreover, the protein expression of FUBP1 in pediatric brain cancer was found to differ across different races, showing high expression in Asian children compared with African-American and Caucasian children (*p* < 0.001).

The genetic instability of cancer cells can lead to a large number of mutations and abnormal activity in certain signaling cascades (Jeggo et al., 2016; Grobner et al., 2018; Zhu et al., 2020). *FUBP1* mutation was found in most of the cancer types analyzed in TCGA, especially in glioma, melanoma, and uterine corpus endometrial carcinoma. Missense mutation is the most important type of mutation observed in *FUBP1*, and R430 in the KH_4 domain is a key site. Moreover, alterations in FUBP1 were found to be closely related to overall survival in pan-cancer according to TCGA datasets. Patients with *FUBP1* alterations had a significantly higher overall survival than those without alterations. However, no difference was found in terms of progression-free survival, which may have resulted from the frequency and means of evaluation as well as the selection of the primary efficacy endpoint.

DNA methylation is considered to be abnormal and can prevent gene activation in various cancer cells (Klutstein et al., 2016; Barwick et al., 2018; Saghafinia et al., 2018; Zafon et al., 2019; Kohler and Rodriguez-Paredes, 2020). Increasing evidence has shown that this stable epigenetic mechanism works in a programmed manner and contributes to the inhibition of cell differentiation processes (Lu et al., 2012; Li et al., 2020). Our analysis of the DNA methylation of *FUBP1* demonstrated definitively low or no methylation in most cancer types analyzed in TCGA, except for UVM and PADD. This finding could verify our previous results indicating enhanced expression of *FUBP1* in most cancers from another perspective.

Regarding the phosphorylation of FUBP1, significant elevation was found in breast cancer at Y126 and T174, in UCEC at T153 and S630, in lung adenocarcinoma at S120, and in clear cell RCC at S120 based on the CPTAC datasets. For the above cancer types, kinases for specific phosphorylation sites could be regarded as efficient therapeutic targets. Moreover, the phosphorylation of FUBP1 at S120 was found to be higher in Asian children than in African-American and Caucasian children, suggesting radial differences in protein phosphorylation levels among individuals with the same disease.

Immunotherapy activates the host immune system to attack cancer cells, and the development of checkpoint blockade antibodies, adoptive T cell therapies, and tumor vaccines has benefitted many cancer patients (Yang, 2015; Peng et al., 2019; Rogado et al., 2019; Truffi et al., 2019; Singh and McGuirk, 2020). In this study, we investigated the correlation of *FUBP1* with various immune cells, including neutrophils, macrophages, fibroblasts, and CD8^+^ T cells. We found that the infiltration of CD8^+^ T cells exhibited a negative correlation with the expression of *FUBP1* in GBM, HNSC-HPV-, and UCEC but either a negative or positive correlation in LGG when different algorithms were used. Moreover, the infiltration of macrophages, neutrophils and cancer-associated fibroblasts was positively correlated with the expression of *FUBP1* with at least two different algorithms. The above findings indicate that the expression of *FUBP1* is associated with the patterns of infiltration of immune cells in various cancers, which may lead to immune evasion.

GO and KEGG enrichment analyses revealed the close relationship of FUBP1 with RNA splicing and the Cdc5L (cell division cycle 5-like protein) complex in all cancer types. Pre-mRNA splicing is essential for most eukaryotic genes, and defects in RNA alternative splicing, including the altered pre-mRNA expression and genetic alterations, have been reported to be closely related to the occurrence and development of various human cancers (Zhang et al., 2019). A large number of candidate biomarkers and potential targets for tumor treatments have been reported based on different RNA splicing patterns (Urbanski et al., 2018; Bonnal et al., 2020). Interestingly, Cdc5L is a pre-mRNA splicing factor consisting of Prp19/Pso4, Plrg 1 and Spf27 that modulates the expression of genes involved in mitosis and the ATR-mediated cell cycle checkpoint (Zhang et al., 2009; Mu et al., 2014). This transcription factor has been reported to promote tumorigenesis in various cancers, such as bladder cancer, ovarian cancer, prostate cancer, and multiple myeloma (Li et al., 2018; Chunmei et al., 2020; Liu et al., 2020; Ziwei et al., 2020). The most enriched GO items suggest the remarkable role of RNA splicing in the mechanism of FUBP1 across different cancer types, and the small-molecule splicing modulators that are currently in clinical trials offer a promising approach to cancer therapy. Signaling pathway analysis demonstrated that alternative splicing and mRNA surveillance were enriched by two different tools, which may contribute to the effect of FUBP1 on tumorigenesis and progression.

## Conclusion

In summary, our first pan-cancer analyses of *FUBP1* revealed remarkable correlations of *FUBP1* with cancer driver events, such as protein phosphorylation, DNA methylation, genetic alteration, and the immune microenvironment. The findings presented in this study contribute to a greater understanding of the function and mechanism of FUBP1 from multiple perspectives based on a large-scale investigation of clinical cancer specimens, and these research efforts support the feasibility of identifying oncogenic drivers in pan-cancer. Further *in vivo* and *in vitro* investigations are required to validate these in silico results.

## Data Availability

The datasets presented in this study can be found in online repositories. The names of the repository/repositories and accession number(s) can be found in the article/ Supplementary Material.
