# Supplementary material for: Pan-Cancer Transcriptome and Immune Infiltration Analyses Reveal the Oncogenic Role of Far Upstream Element-Binding Protein 1 (FUBP1)
Source: Front Mol Biosci. 2022 Feb 22;9:794715. doi: 10.3389/fmolb.2022.794715 (PMC8902172; doi:10.3389/fmolb.2022.794715)
Supplement: Supplementary file 1 [file DataSheet2.PDF]

Supplementary Material

Supplementary Figures and Tables

1. Supplementary Figures

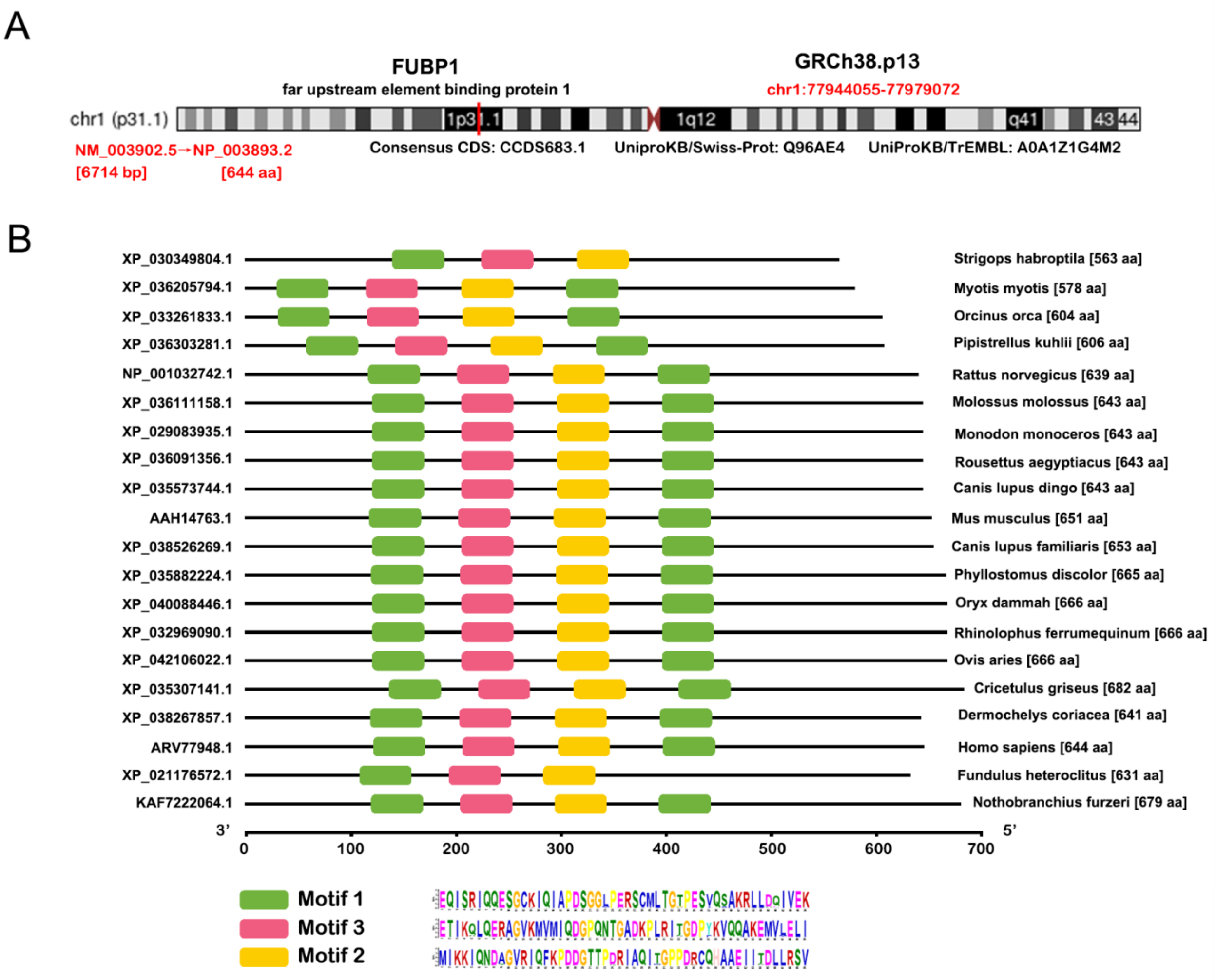

Supplementary Figure 1. Structural characteristics of FUBP1 in different species. (A) Genomic location of FUBP1 in human sapiens. (B) Conserved motifs of the FUBP1 protein among different species.

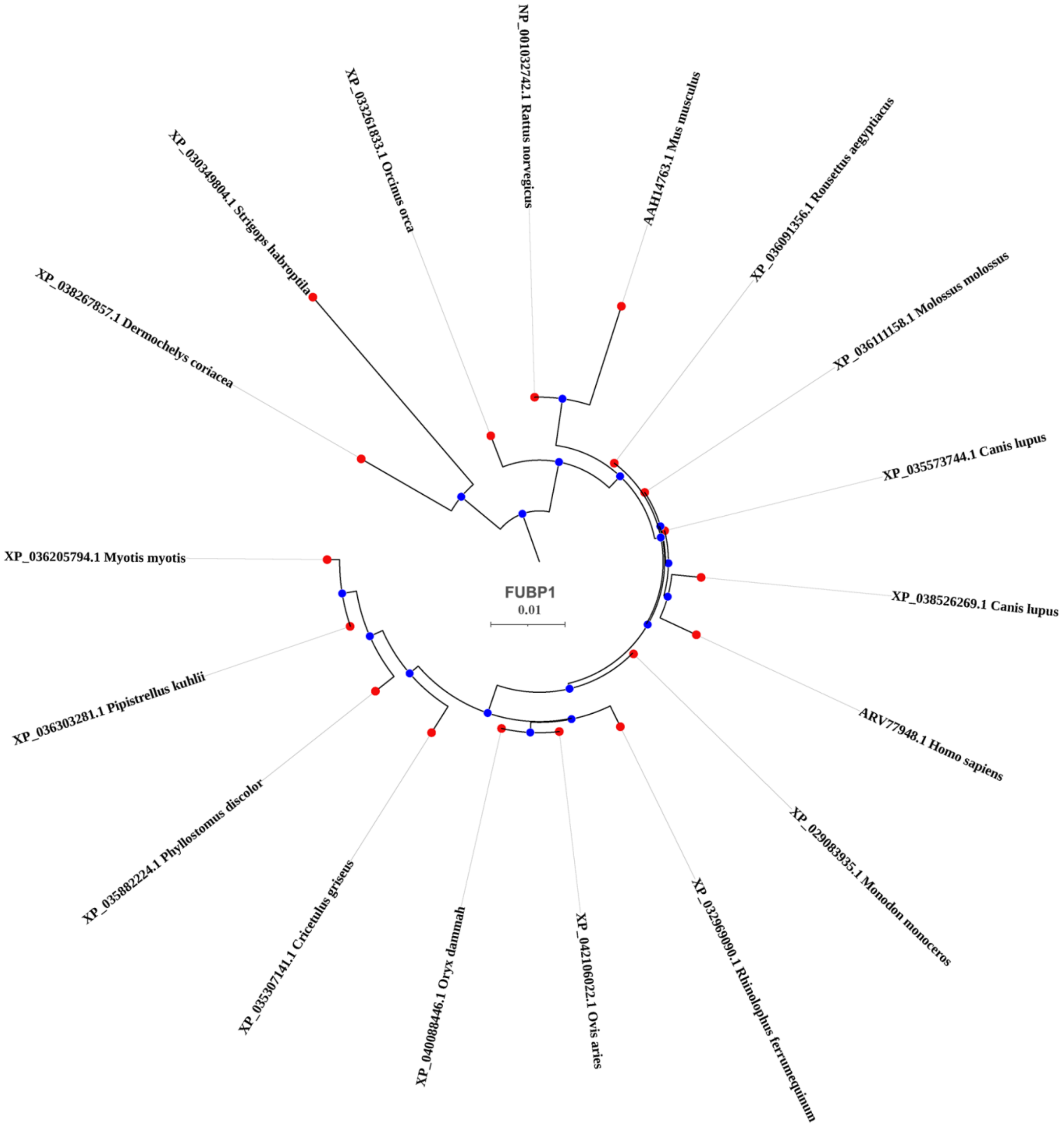

Supplementary Figure 2. The phylogenetic tree of FUBP1 in different species. Molecular Evolutionary Genetics Analysis (MEGA7) was used to align the protein sequences and obtain the phylogenetic tree of FUBP1 in multiple species.

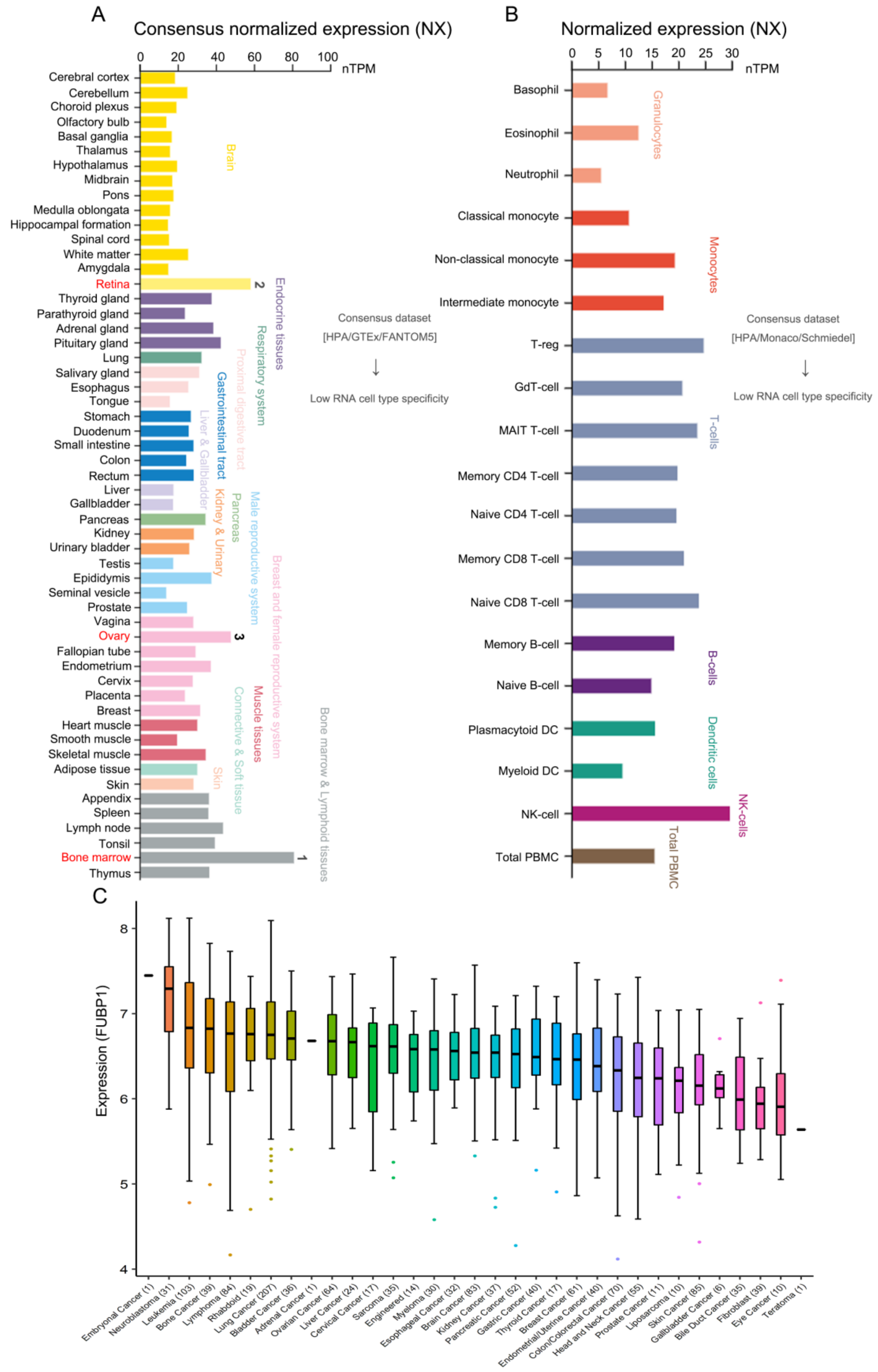

Supplementary Figure 3. Expression of *FUBP1* in different normal tissues and in normal and malignant cells. The expression level of *FUBP1* in different tissues was investigated using HPA, FANTOM5, and GTEx (A) or in various immune cells using datasets in HPA, Schmiedel, and Monaco (B). (C) Transcriptome data of *FUBP1* in various tumor cell lines based upon data generated by Cancer Cell Line Encyclopedia (CCLE).

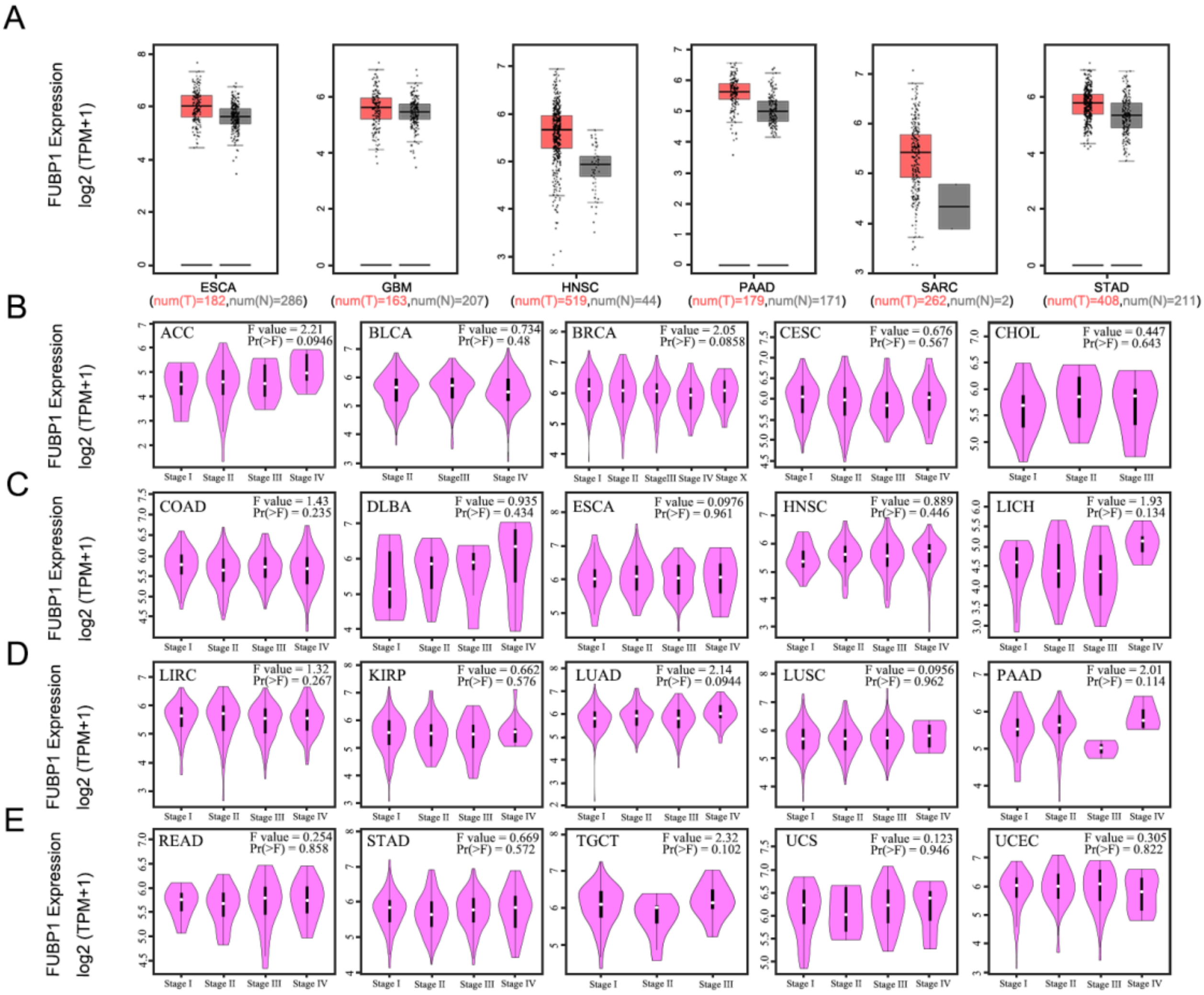

Supplementary Figure 4. Expression level of the *FUBP1* gene in different cancer types and clinical pathological stages. (A) The expression level of *FUBP1* in ESCA, GBM, HNSC, PAAD, SARC, STAD and their normal tissues in TCGA database. The expression levels of FUBP1 in different clinical stages of ACC, BLCA, BRCA, CESC, CHOL (B); COAD, DLBA, ESCA, HNSC, LIHC (C); LIRC, KIRP, LUAD, LUSC, PAAD (D); READ, STAD, TGCT, UCS, UCEC (E) were quantified with log2 (TPM+1) and visualized by GEPIA2.

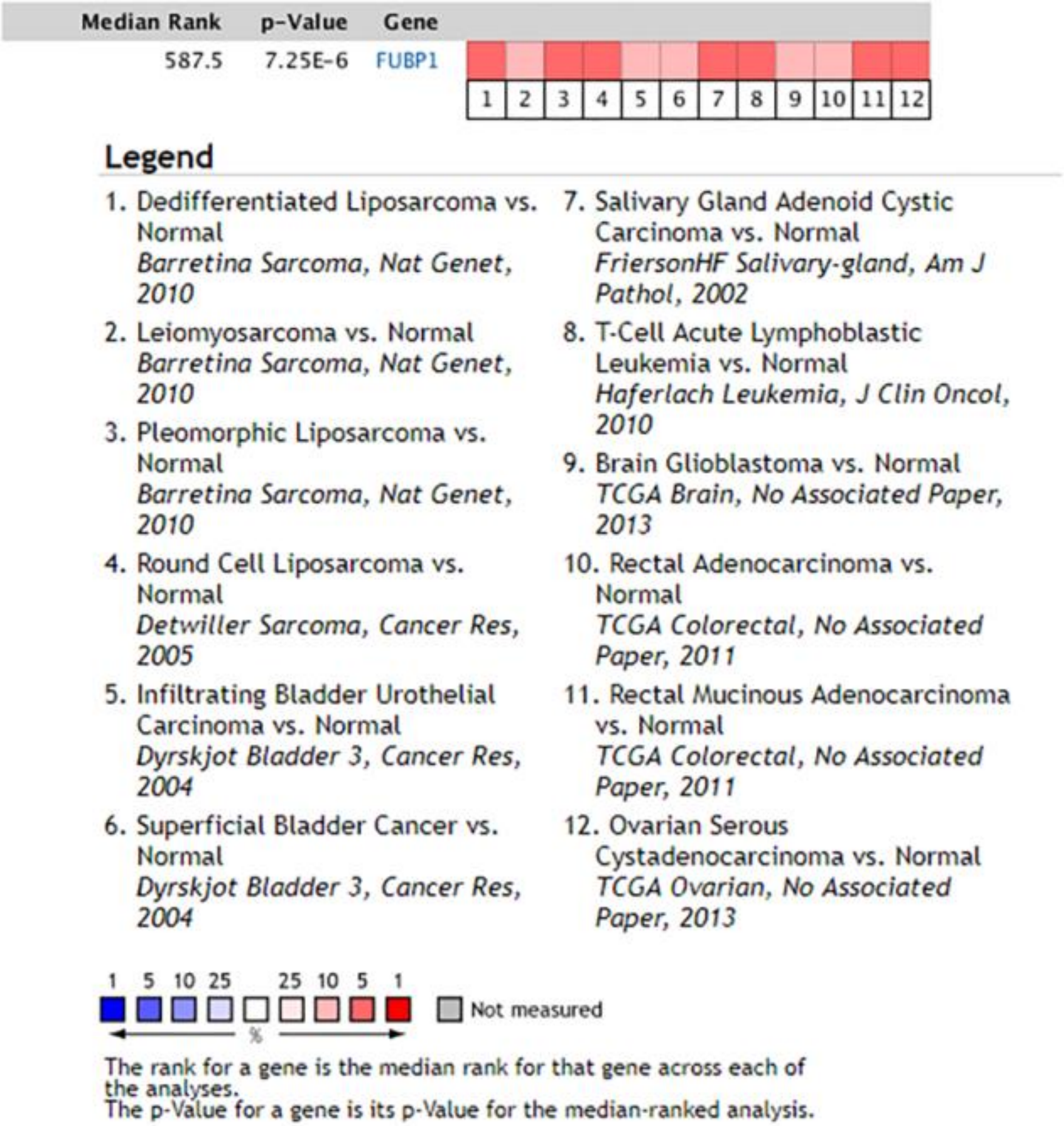

Supplementary Figure 5. Meta-analysis of the expression of *FUBP1* between cancer and adjacent normal tissues. The expression of *FUBP1* in liposarcoma, leiomyosarcoma, bladder urothelial carcinoma, salivary gland adenoid cystic carcinoma, T-cell acute lymphoblastic leukemia, brain glioblastoma, rectal adenocarcinoma, rectal mucinous adenocarcinoma, and ovarian serous cystadenocarcinoma was explored using the Oncomine database.

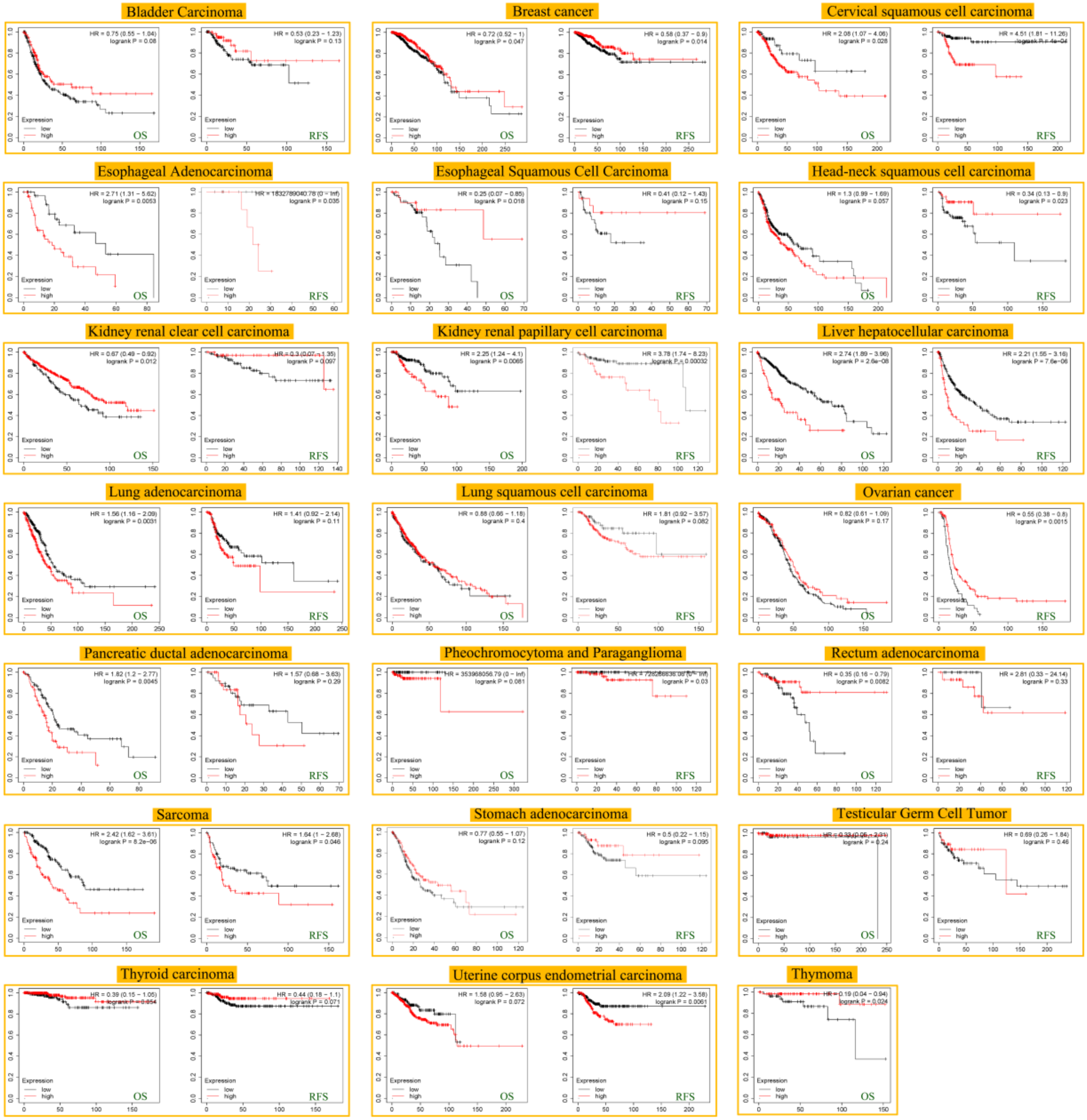

Supplementary Figure 6. Correlation between *FUBP1* expression and clinical prognosis in different cancers. Survival analyses, including OS and RFS, were both performed in pan-cancer using Kaplan-Meier plotter.

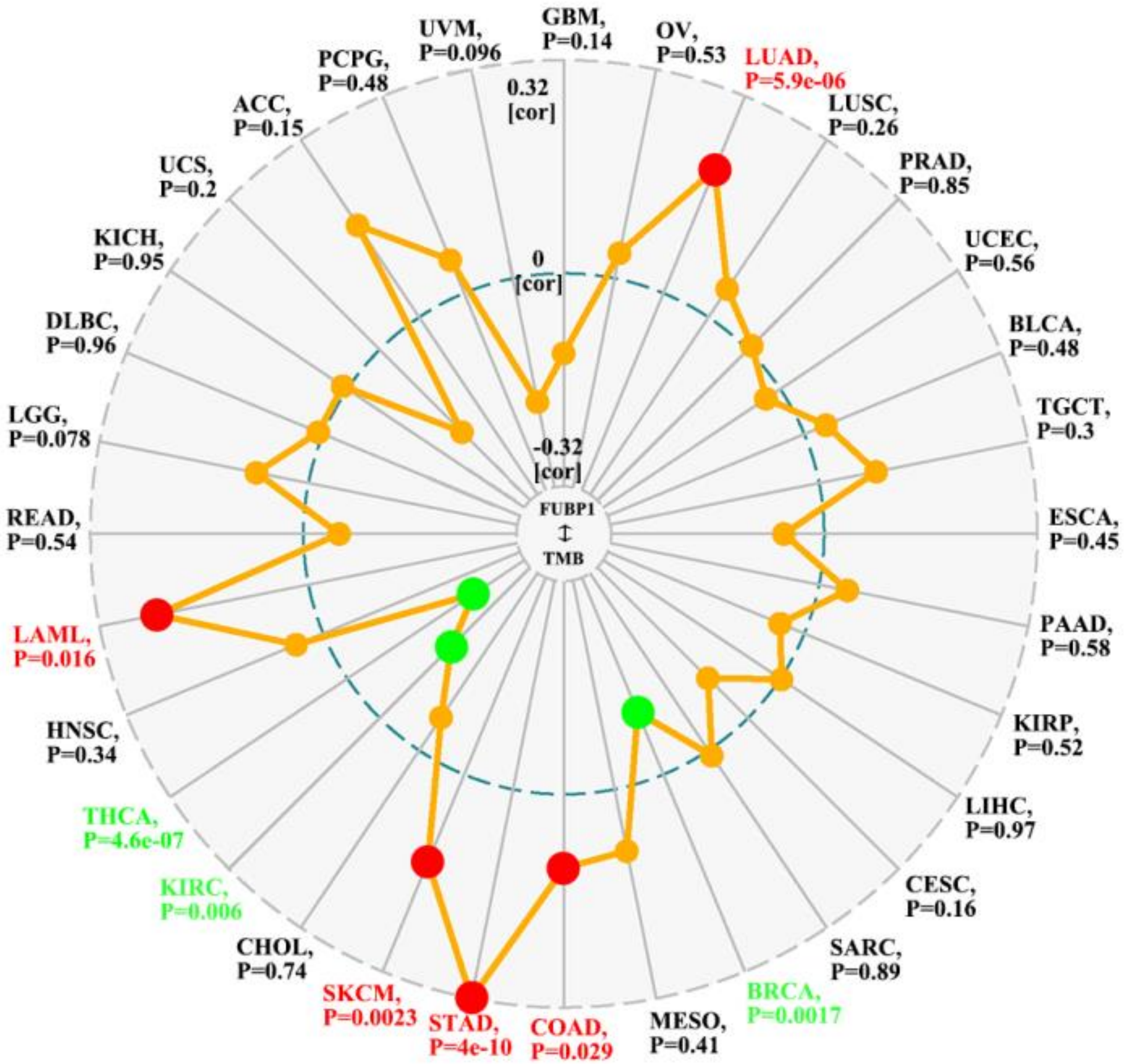

Supplementary Figure 7. Correlation between the expression of *FUBP1* and tumor mutational burden. We explored the correlation between the expression of *FUBP1* and tumor mutational burden in different cancers in TCGA via the website http://sangerbox.com/. The red spots represent a positive correlation, and the green spots represent a negative correlation. Differences were analyzed by Spearman’s rank correlation test, and the *p* values were obtained.

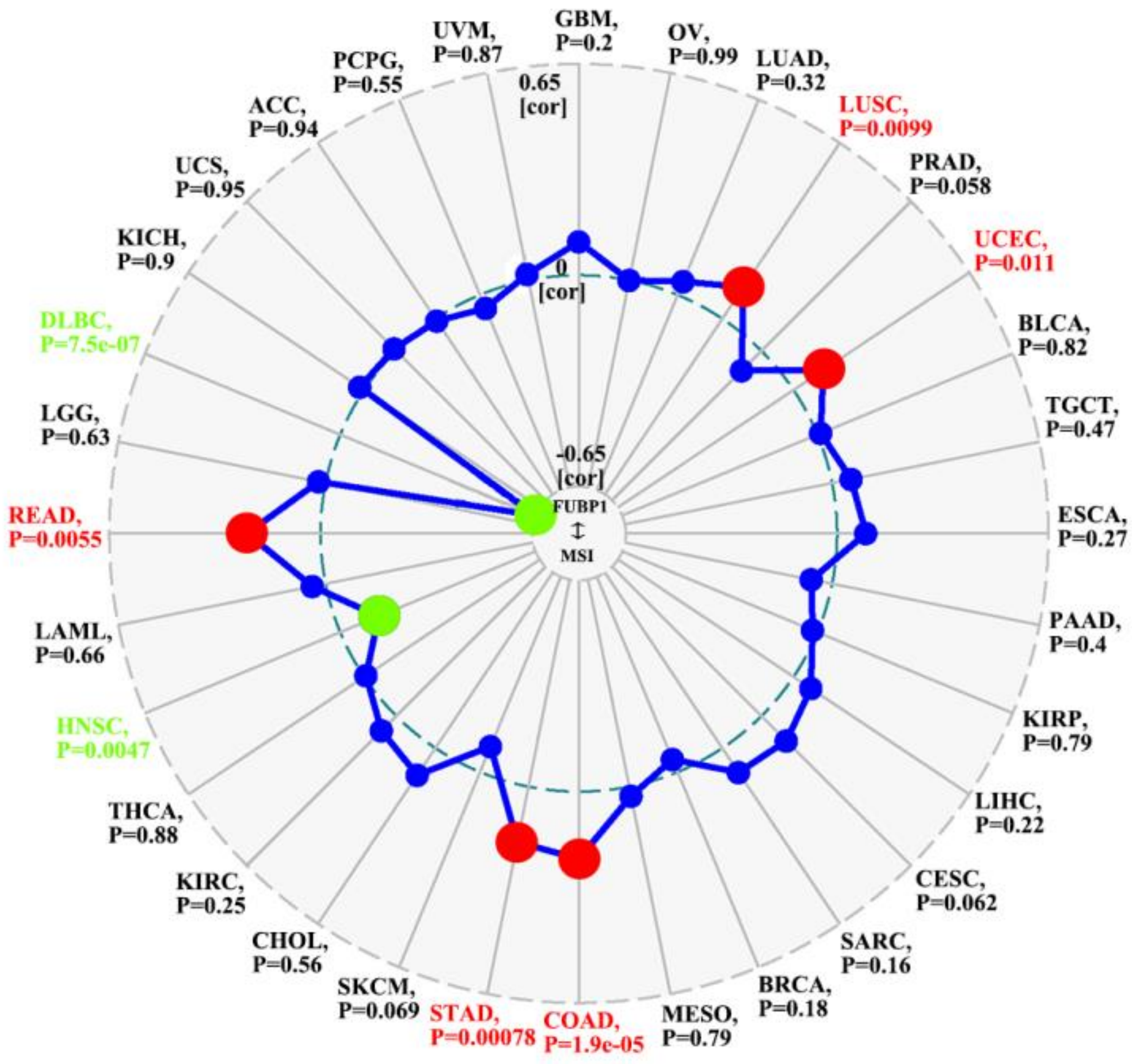

Supplementary Figure 8. Correlations between the expression of *FUBP1* and microsatellite instability in different cancer types. We explored the correlation between the expression of *FUBP1* and microsatellite instability in different cancers in TCGA via the website http://sangerbox.com/. The red spots represent a positive correlation, and the green spots represent a negative correlation. Differences were analyzed by Spearman's rank correlation test, and the *p* values were obtained.

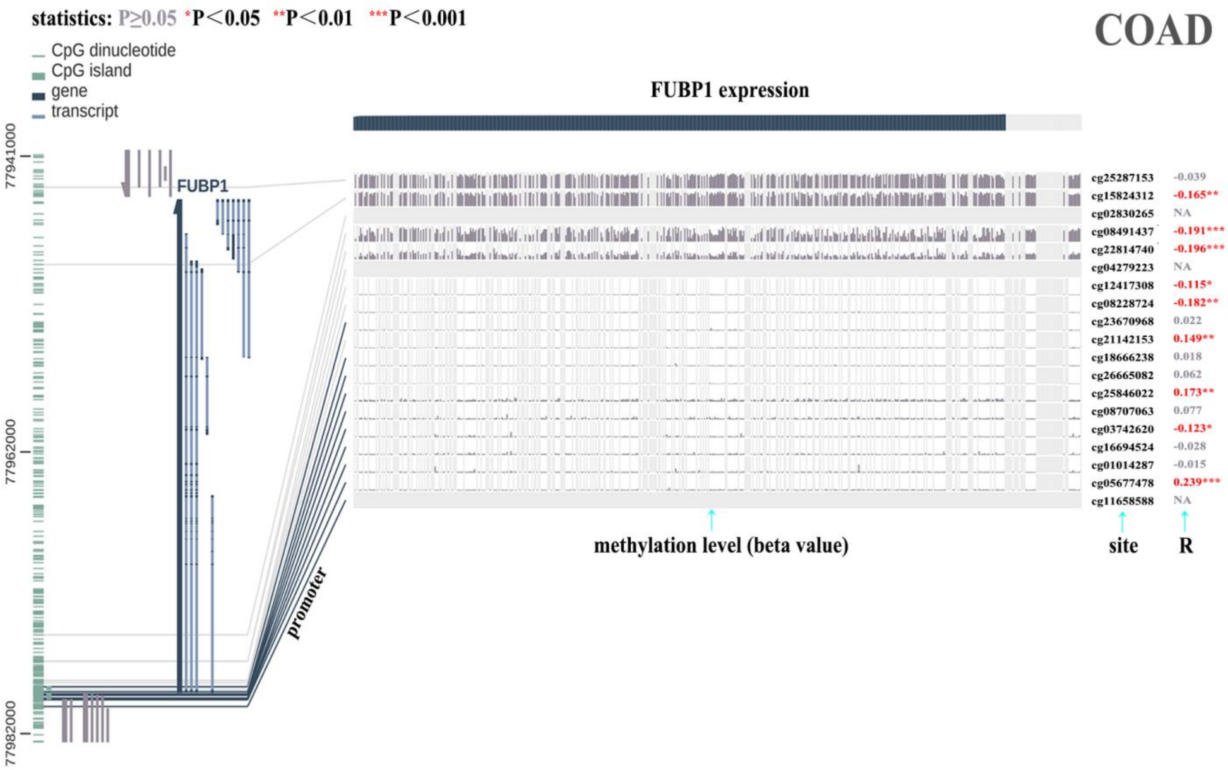

Supplementary Figure 9. Association between the methylation of *FUBP1* and gene expression in COAD cases in TCGA. The DNA methylation level of *FUBP1* with multiple probes was analyzed using the MEXPRESS approach. The beta values that illustrate the methylation state and the Pearson correlation coefficients are displayed. *p<0.05, **p<0.01, ***p<0.001.

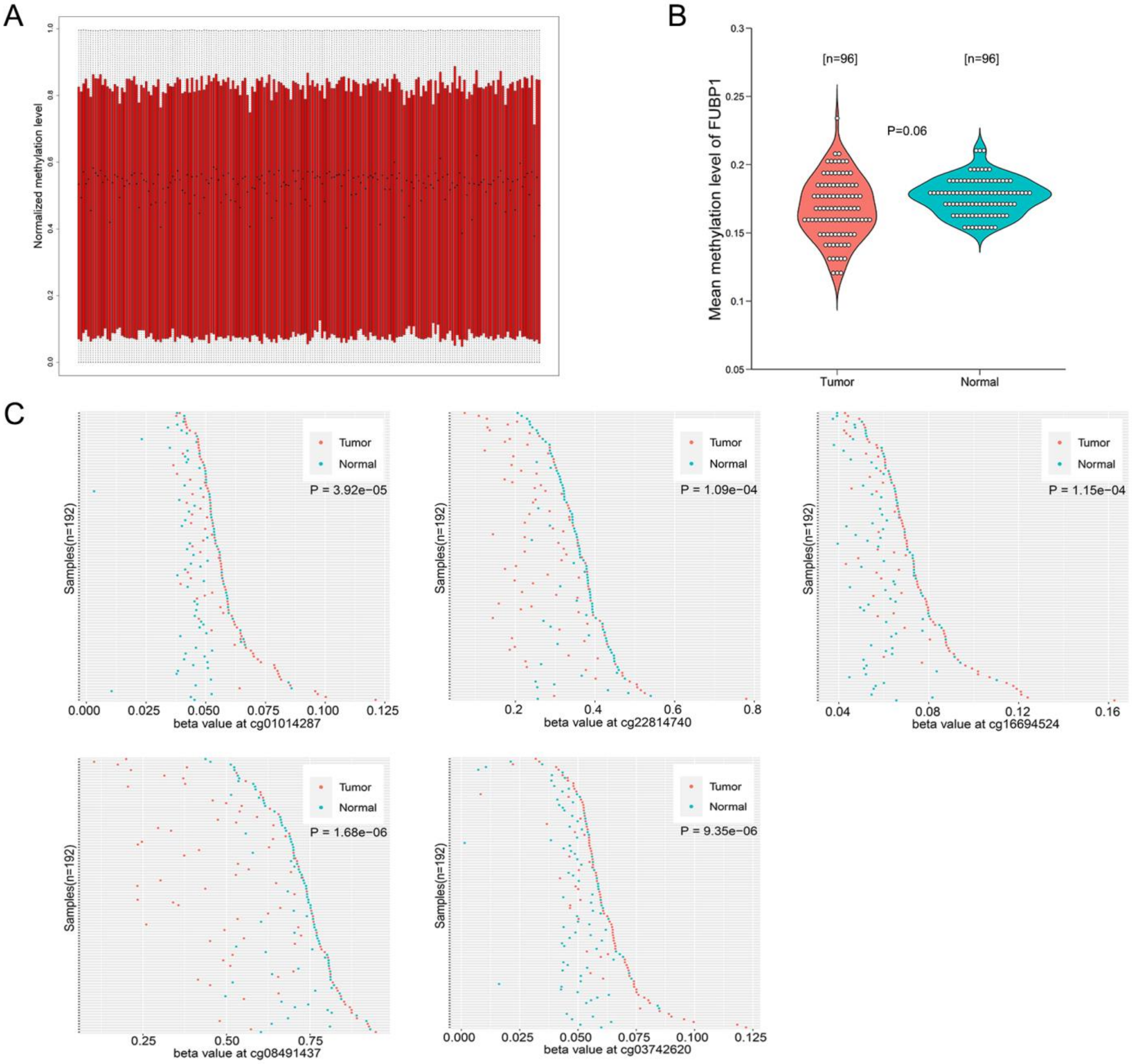

Supplementary Figure 10. DNA methylation status of *FUBP1* in the GSE131013 dataset. (A) Normalization of GSE131013 data; (B) methylation level of *FUBP1* in COAD tissues and adjacent normal tissues; (C) beta value of *FUBP1* with specific probes, including cg22814740 and cg08491437.

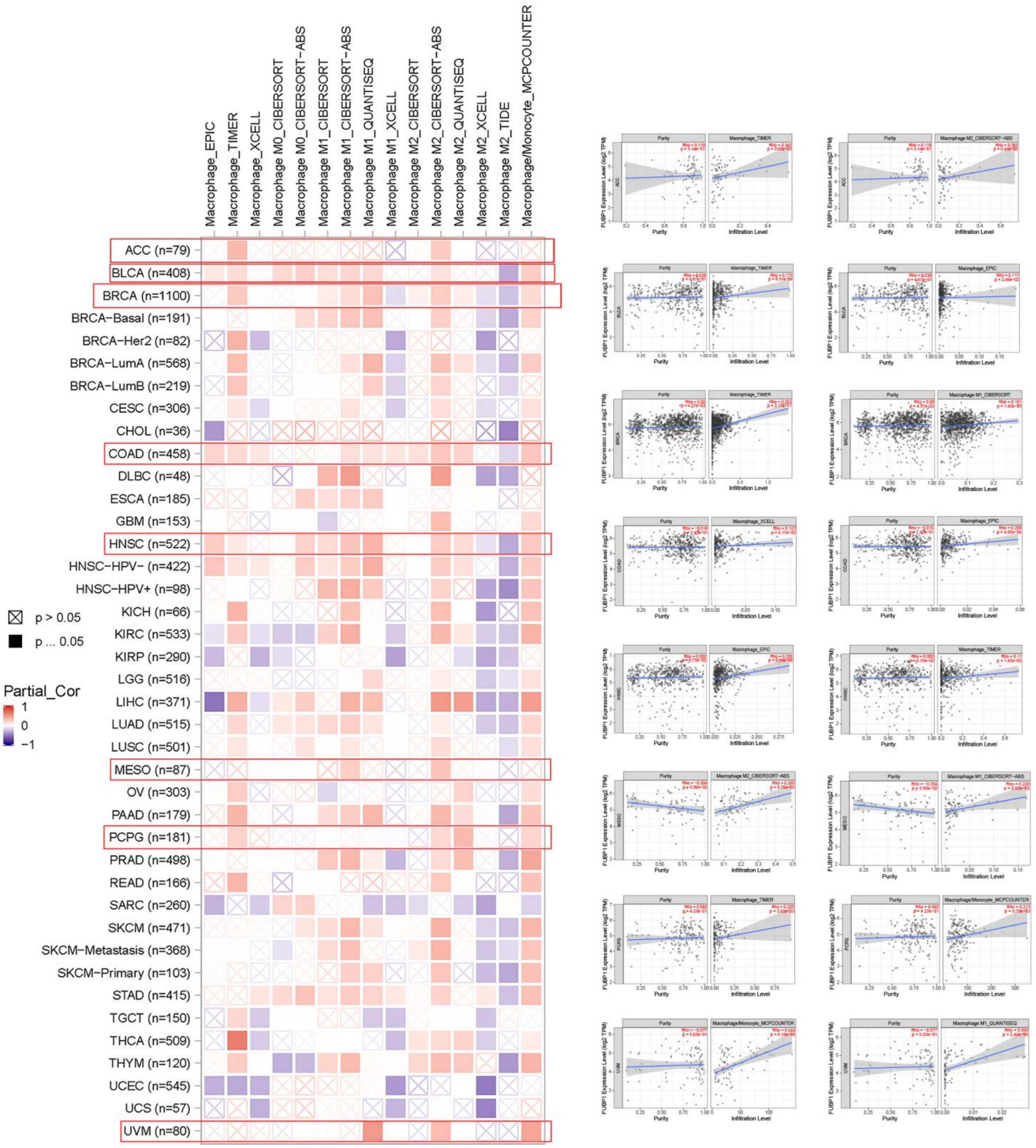

Supplementary Figure 11. Positive correlation between the expression of *FUBP1* and immune infiltration of macrophages. EPIC, TIMER, XCELL, CIBERSORT, QUANTISEQ, TIDE, and MCPCOUNTER were used to explore the correlation between *FUBP1* and the infiltration level of macrophages in different cancers of TCGA.

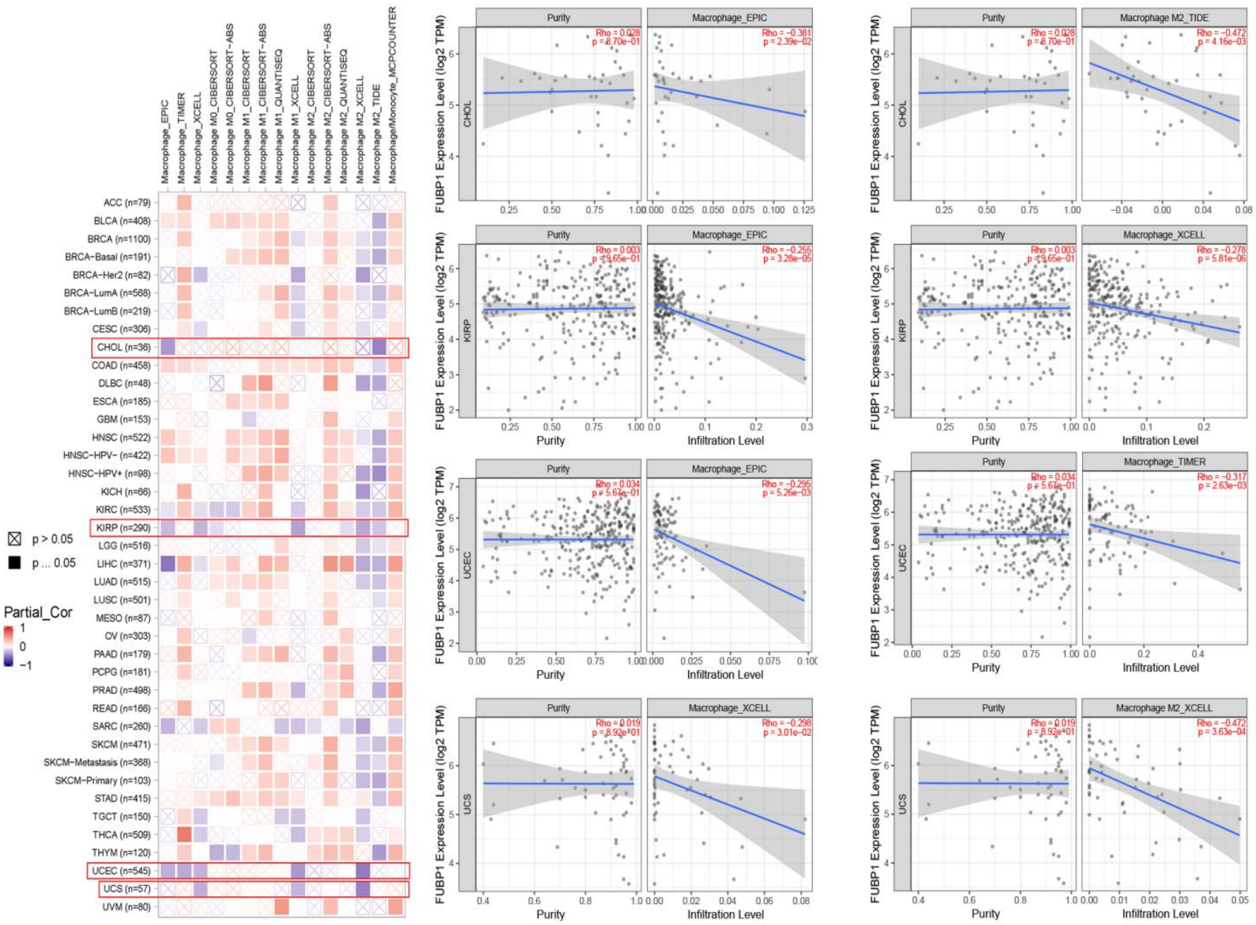

Supplementary Figure 12. Negative correlations between the expression of *FUBP1* and the infiltration level of macrophages. CHOL, KIRP, UCEC, and UCS showed negative correlation with the infiltration of macrophages using at least two different algorithms. P values and Rho values were displayed.

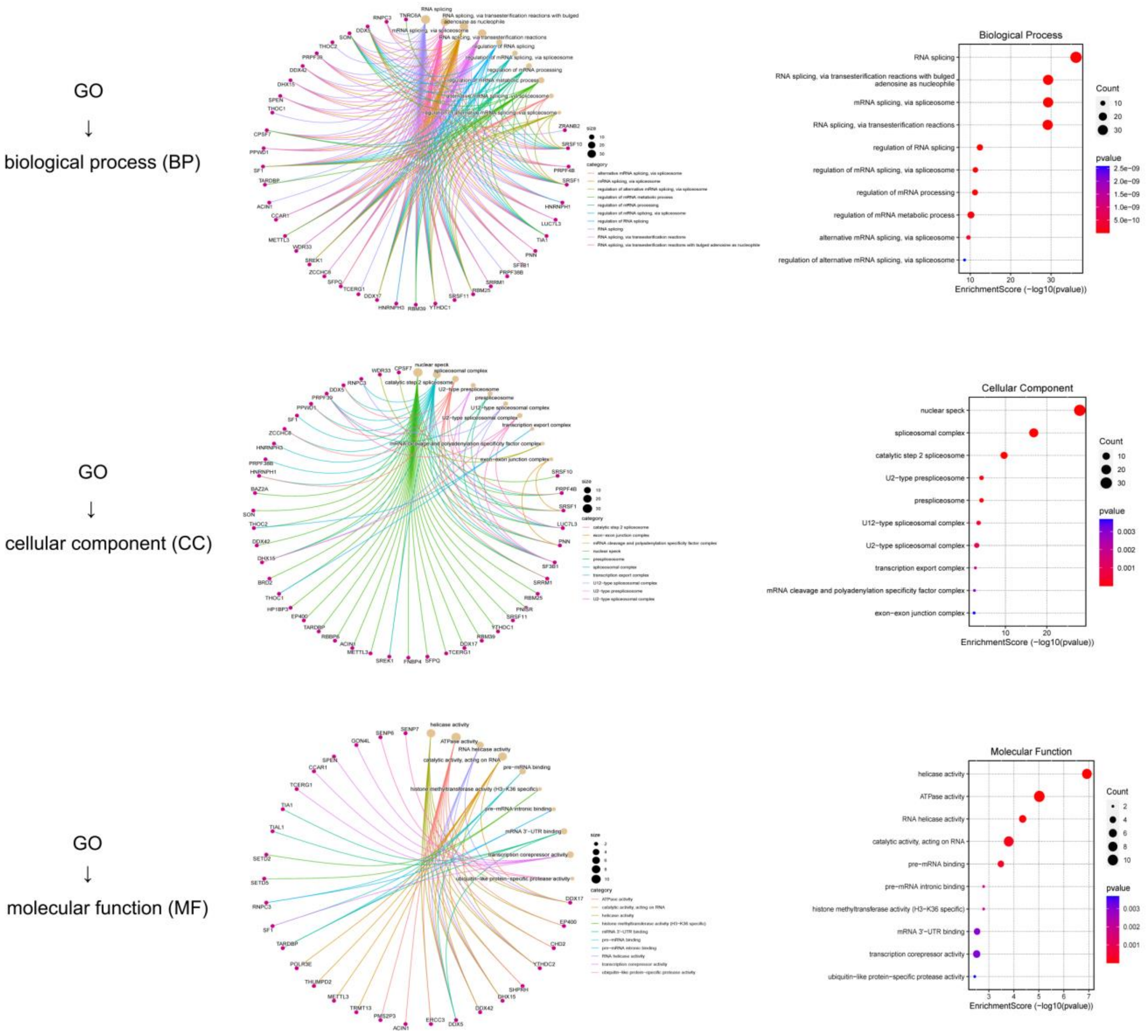

Supplementary Figure 13. GO analysis of FUBP1-related genes in various cancers. Biological process, cellular component, and molecular function were enriched using the "clusterfiler" R package.

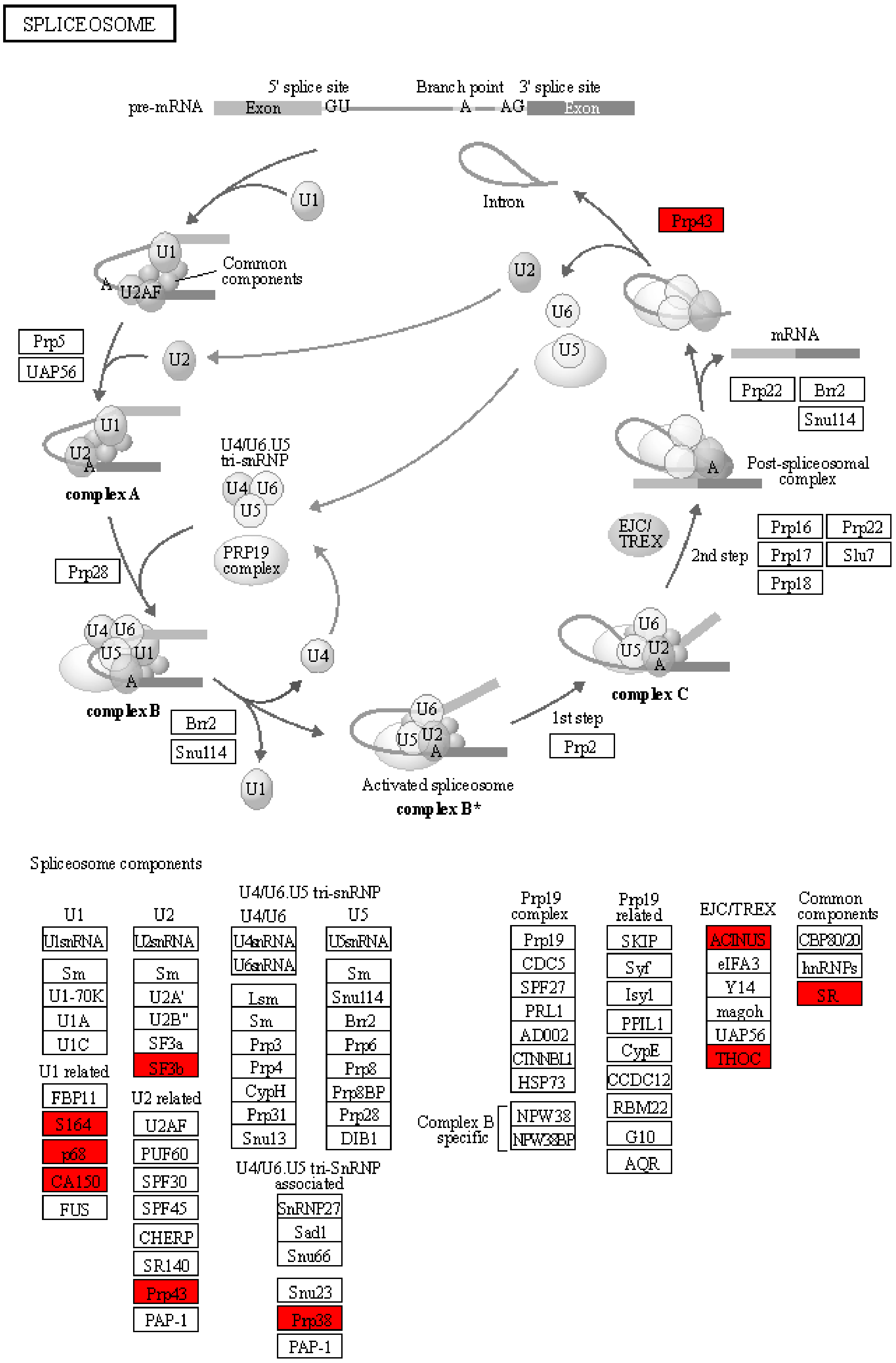

Supplementary Figure 14. Enriched spliceosome pathways and key signaling spots are displayed. Genes in red represent elevated expression. The Pathview R package was used to color the elevated genes.

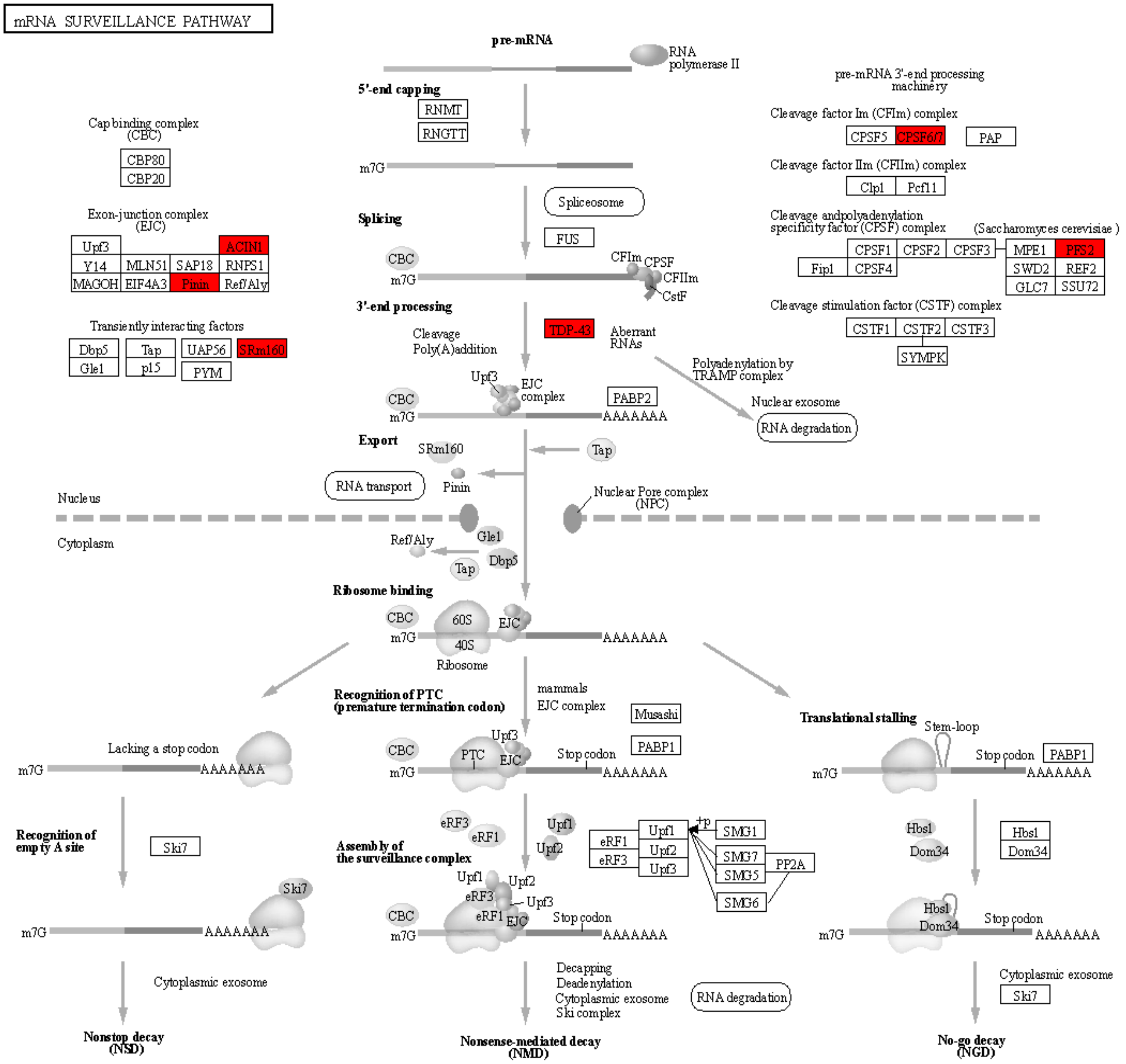

Supplementary Figure 15. Enriched mRNA surveillance pathways and the key spots in the signaling are displayed. Genes in red represent elevated expression. The Pathview R package was used to color the elevated genes.
