# Supplementary figures and images for: Pan-Cancer Transcriptome and Immune Infiltration Analyses Reveal the Oncogenic Role of Far Upstream Element-Binding Protein 1 (FUBP1)
Source: Front Mol Biosci. 2022 Feb 22;9:794715. doi: 10.3389/fmolb.2022.794715 (PMC8902172; doi:10.3389/fmolb.2022.794715)

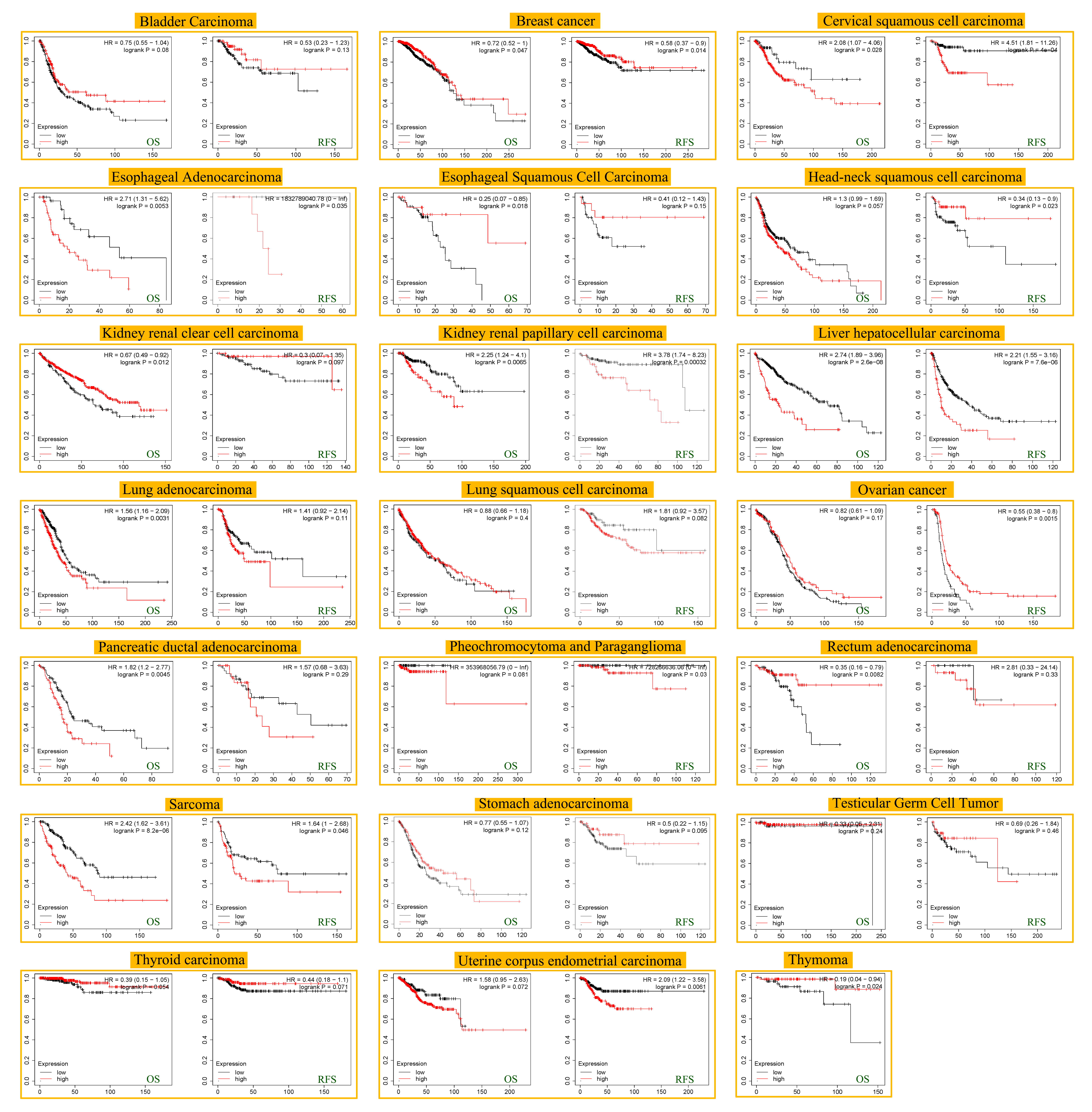

Supplement: Supplementary file 2 [file Image6.TIF]

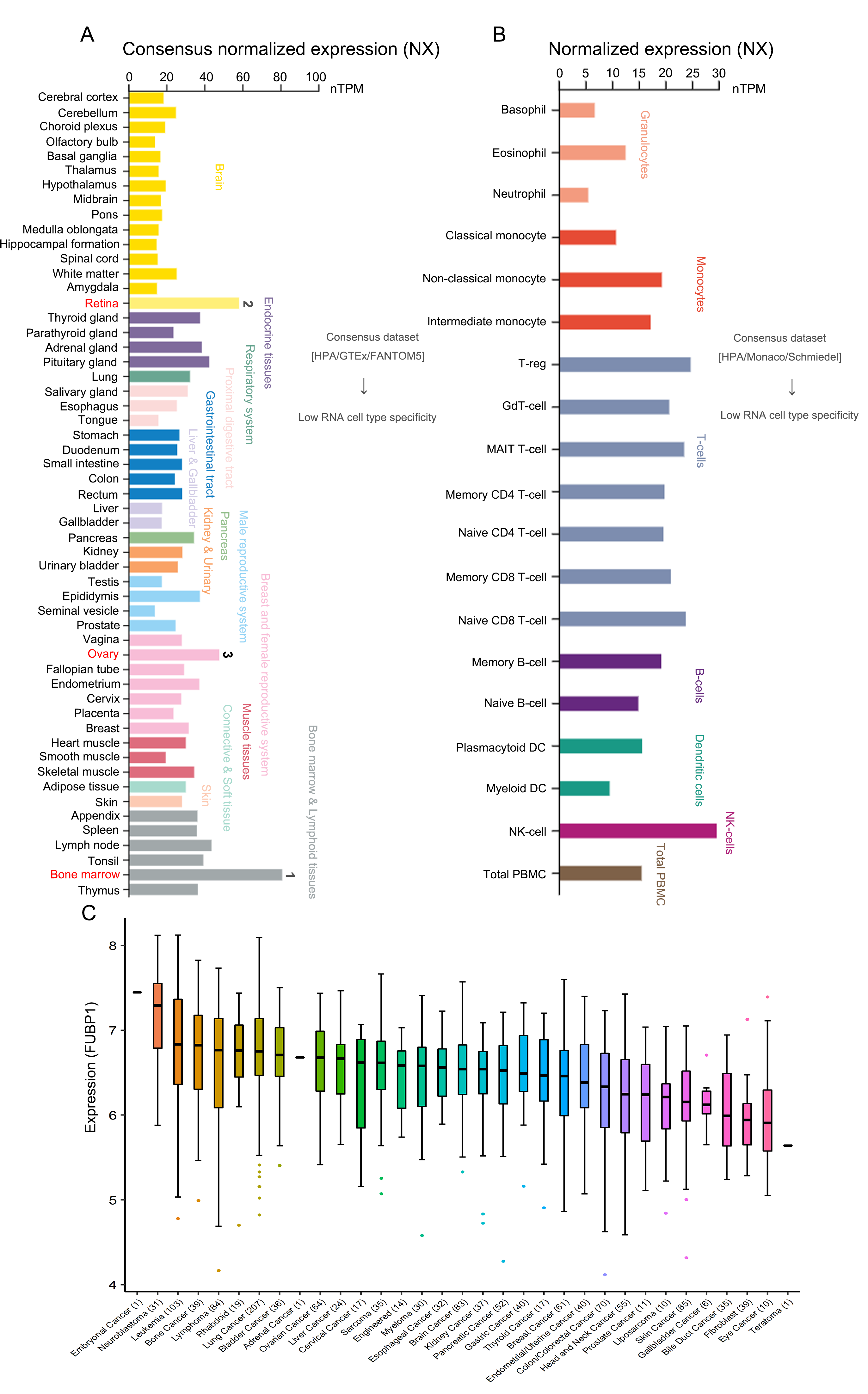

Supplement: Supplementary file 5 [file Image3.TIF]

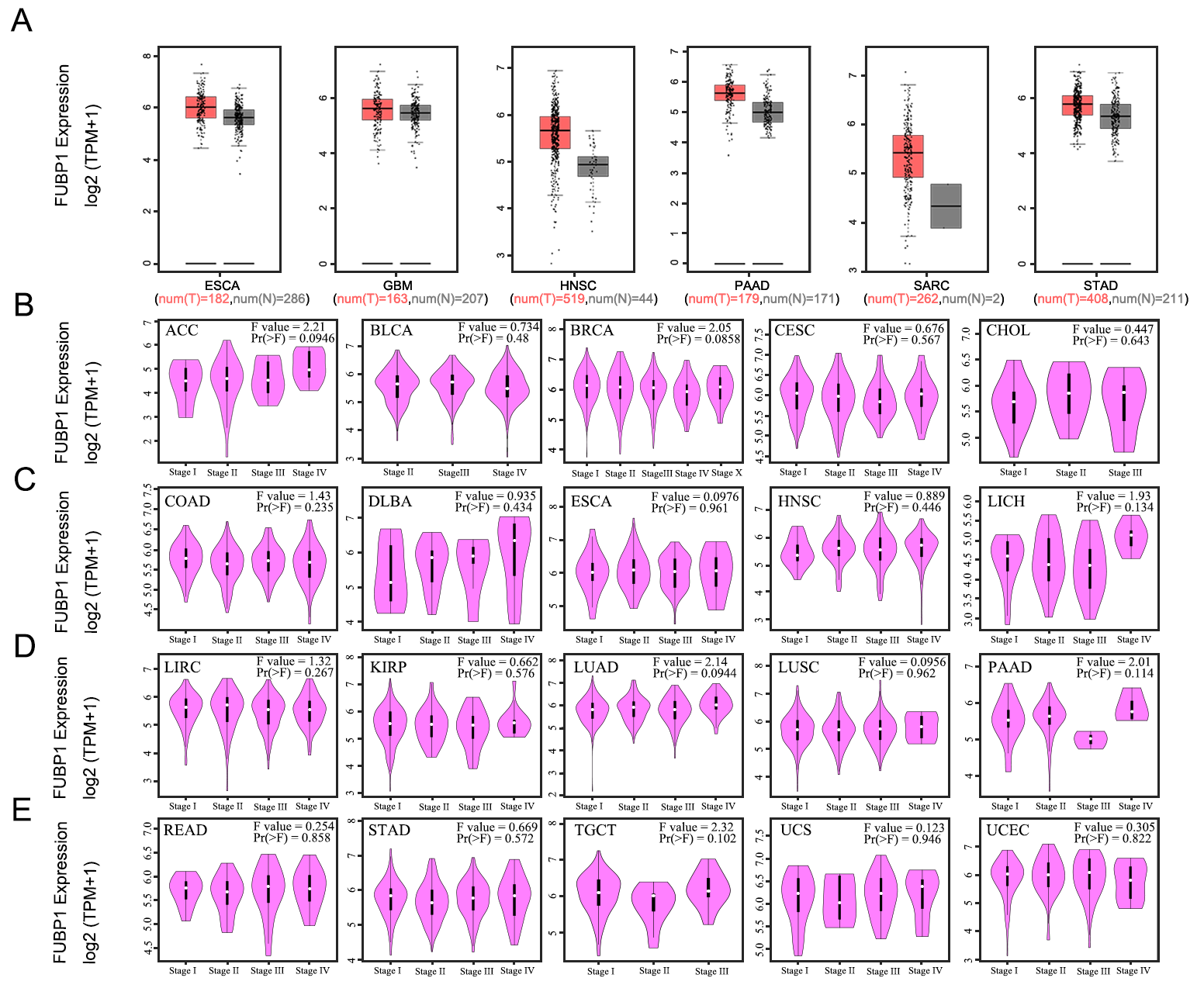

Supplement: Supplementary file 6 [file Image4.TIF]

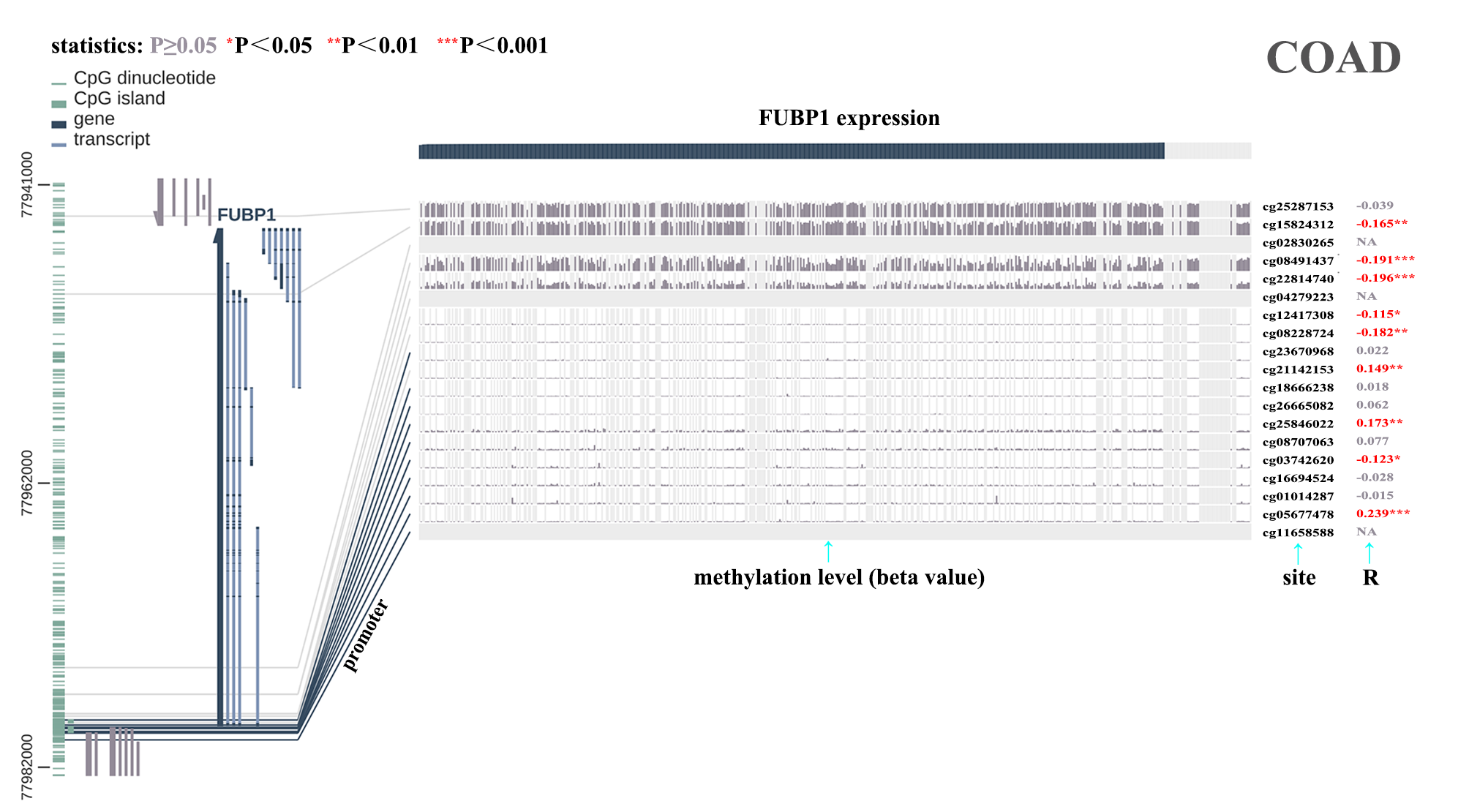

Supplement: Supplementary file 7 [file Image9.TIF]

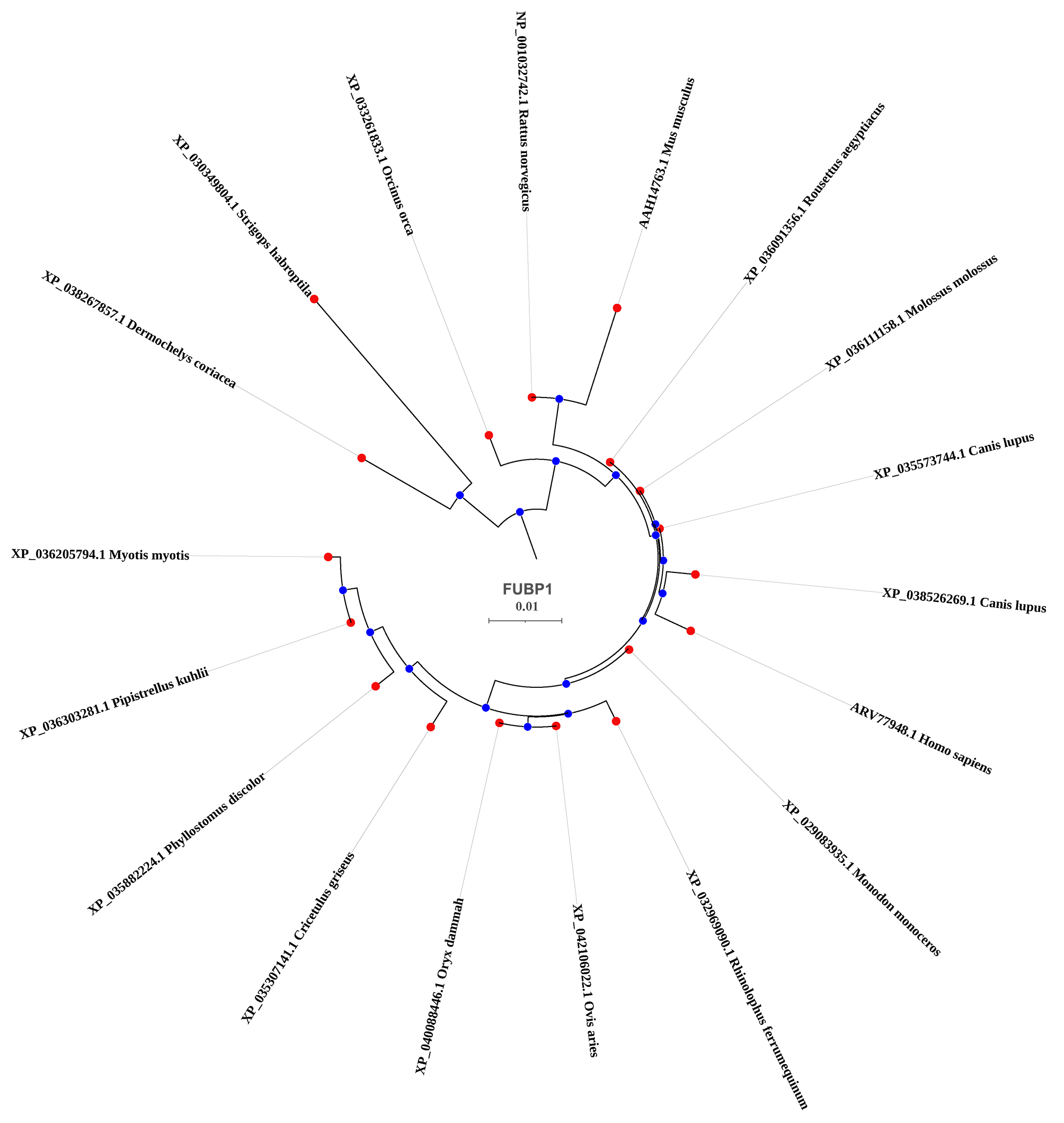

Supplement: Supplementary file 8 [file Image2.TIF]

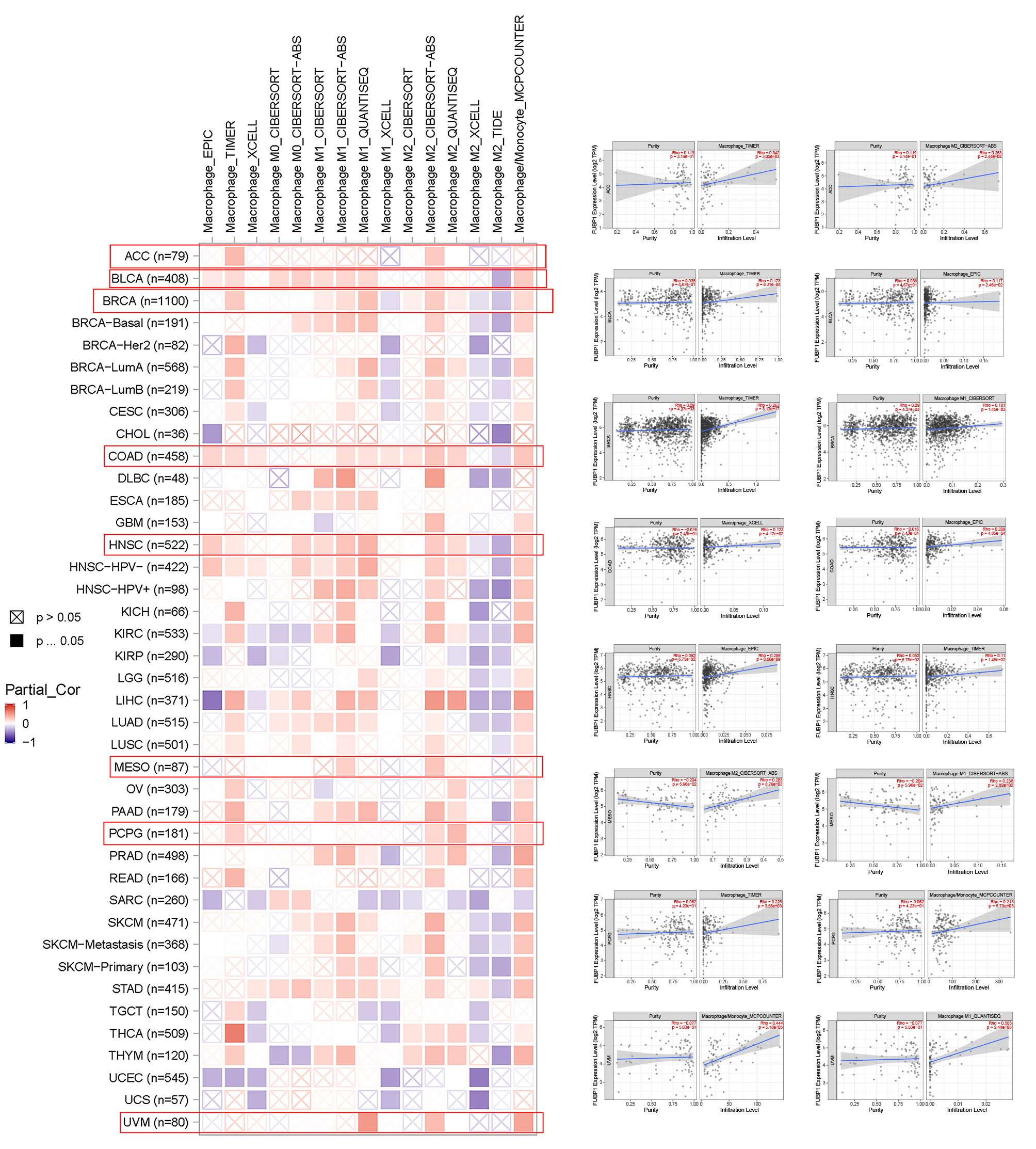

Supplement: Supplementary file 10 [file Image11.TIF]

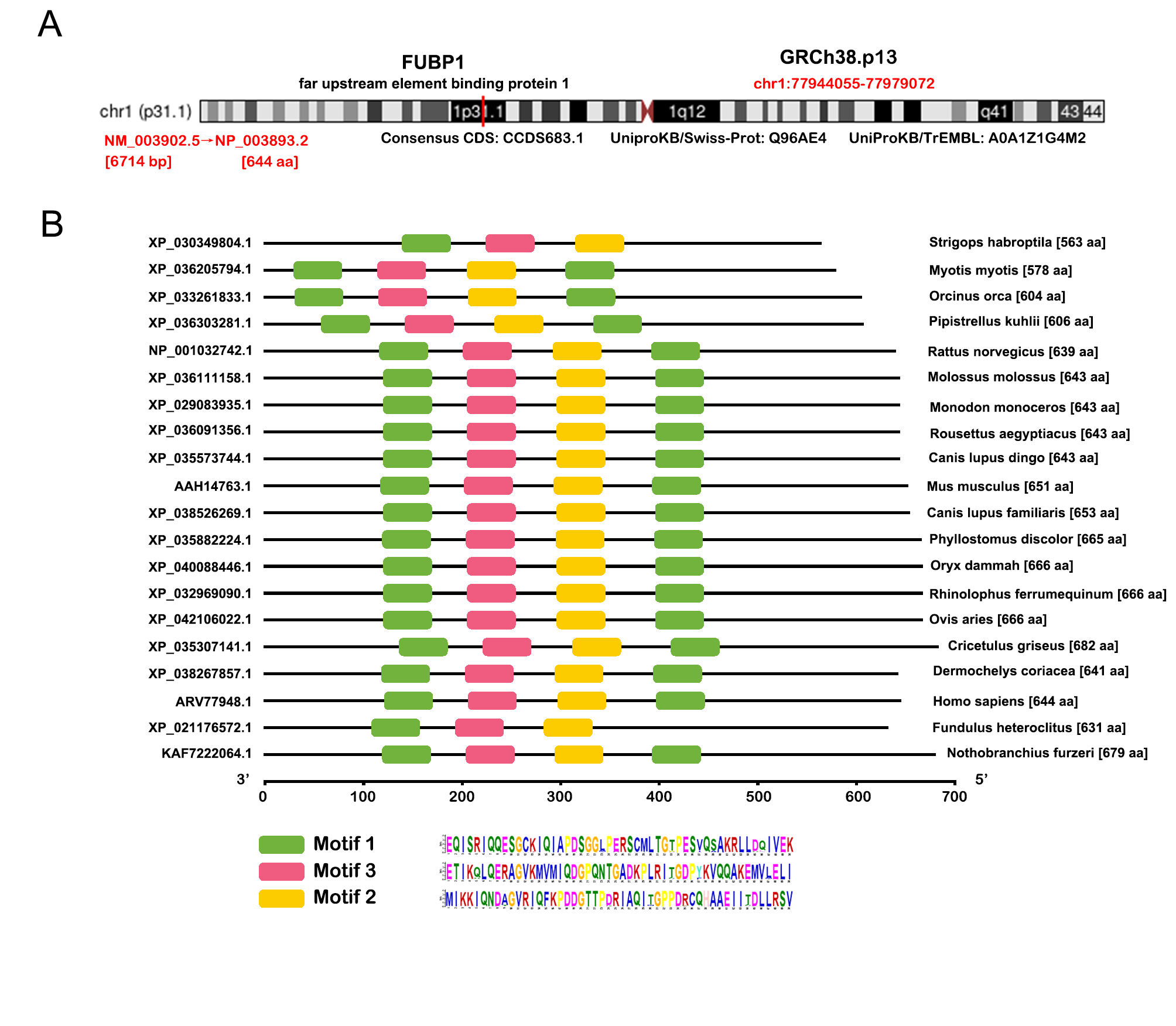

Supplement: Supplementary file 11 [file Image1.TIF]

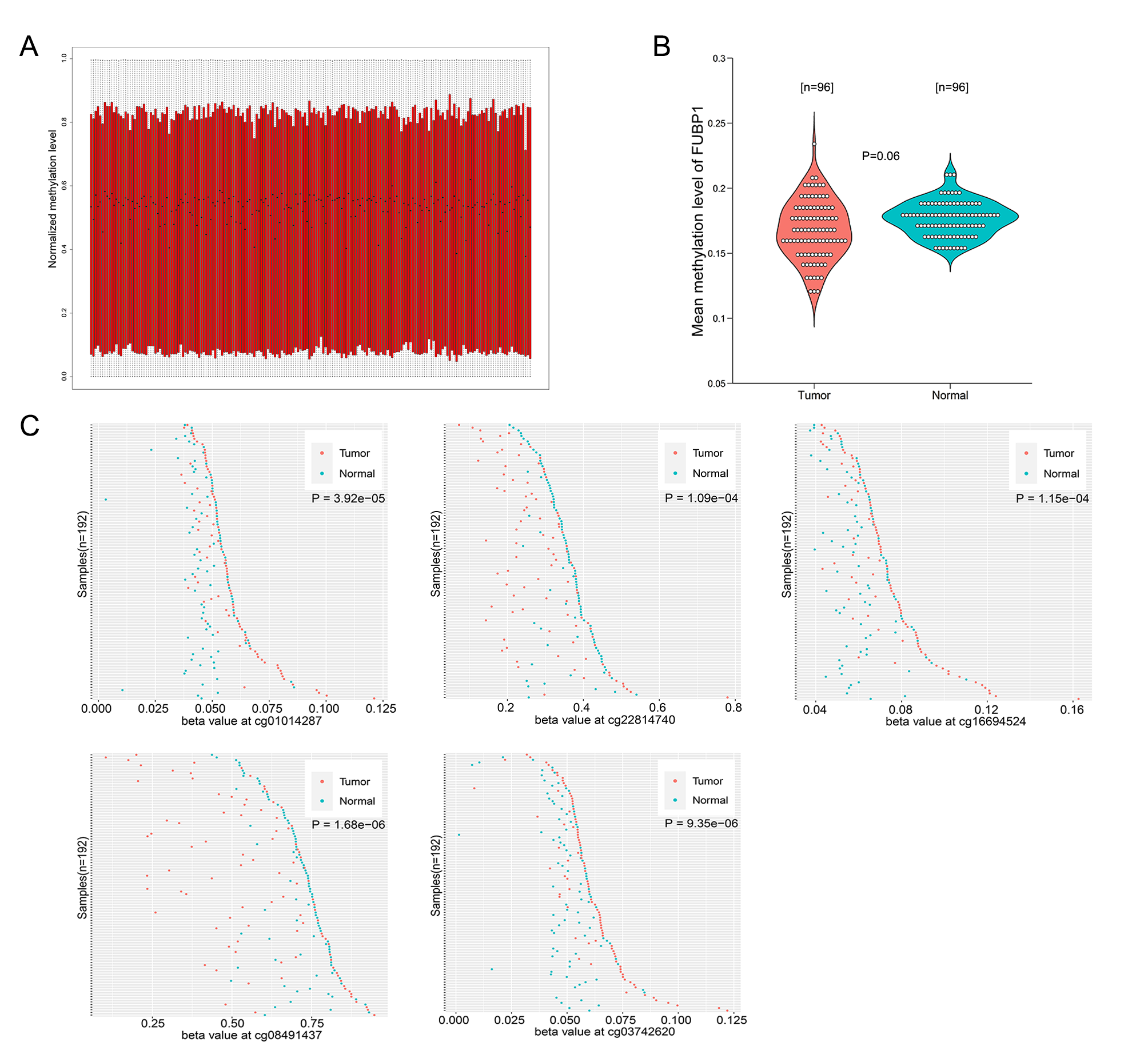

Supplement: Supplementary file 12 [file Image10.TIF]

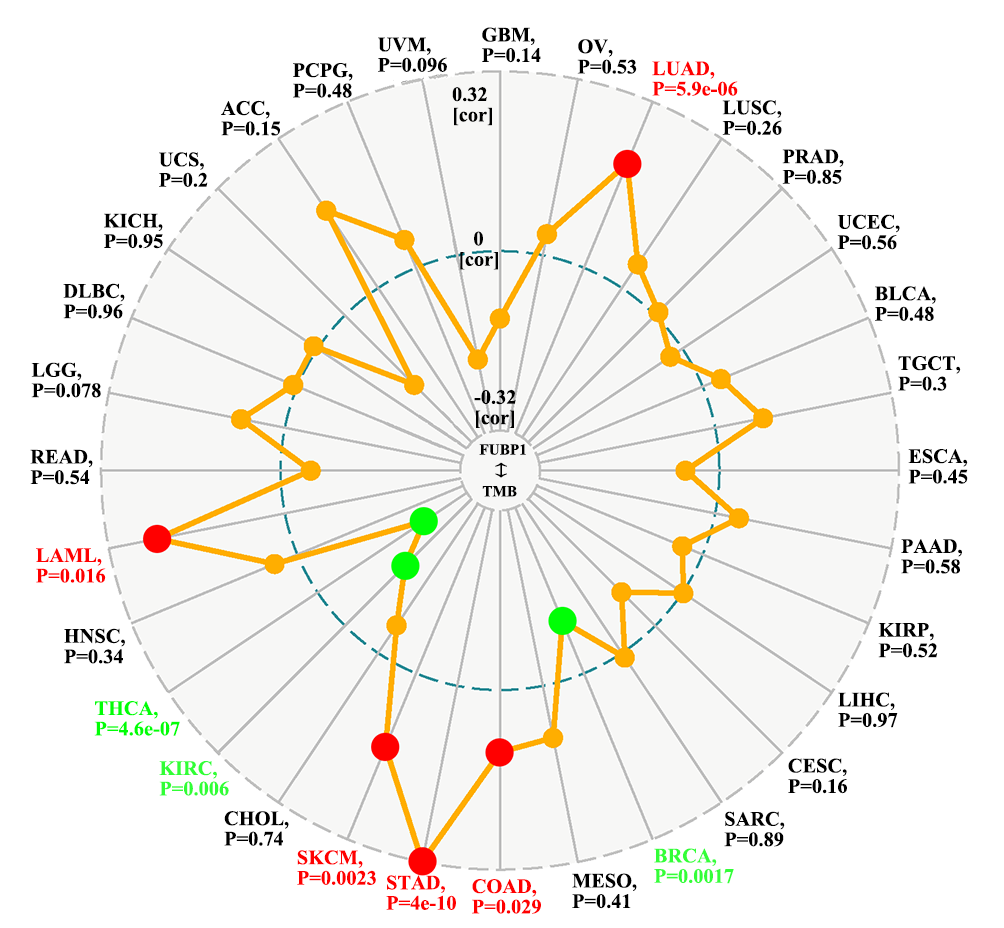

Supplement: Supplementary file 13 [file Image7.TIF]

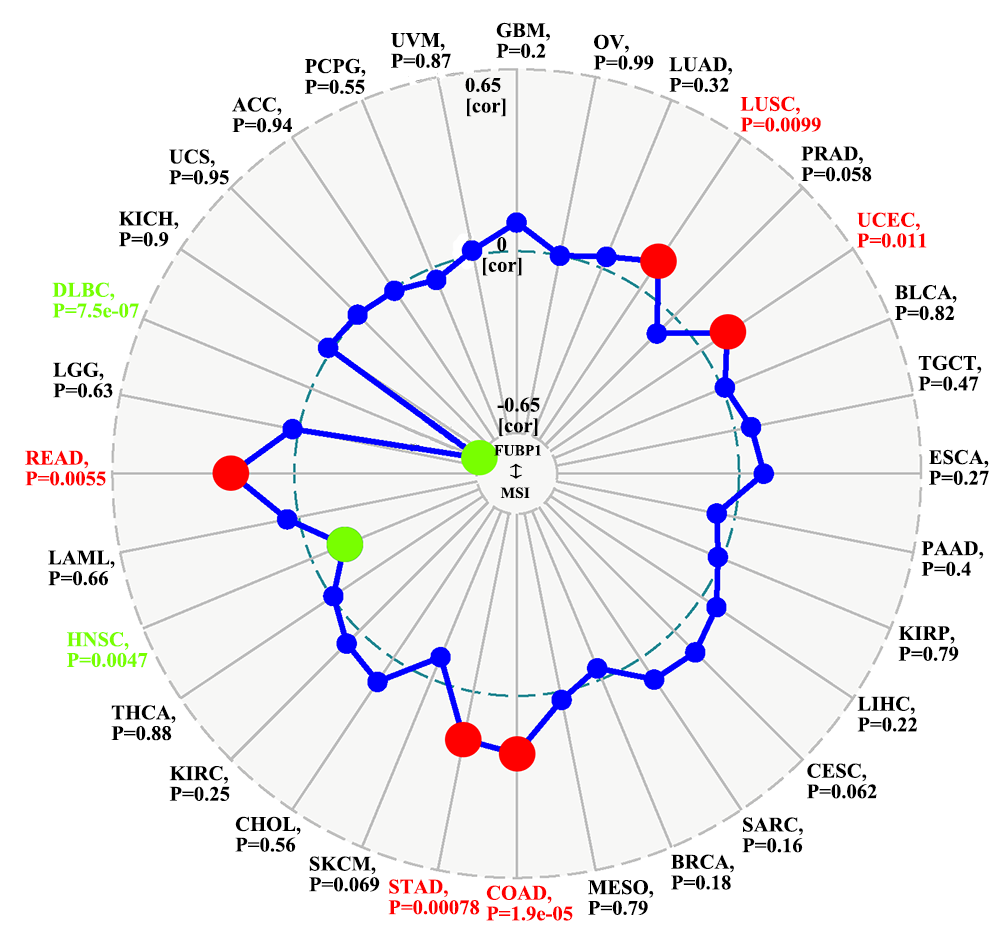

Supplement: Supplementary file 15 [file Image8.TIF]

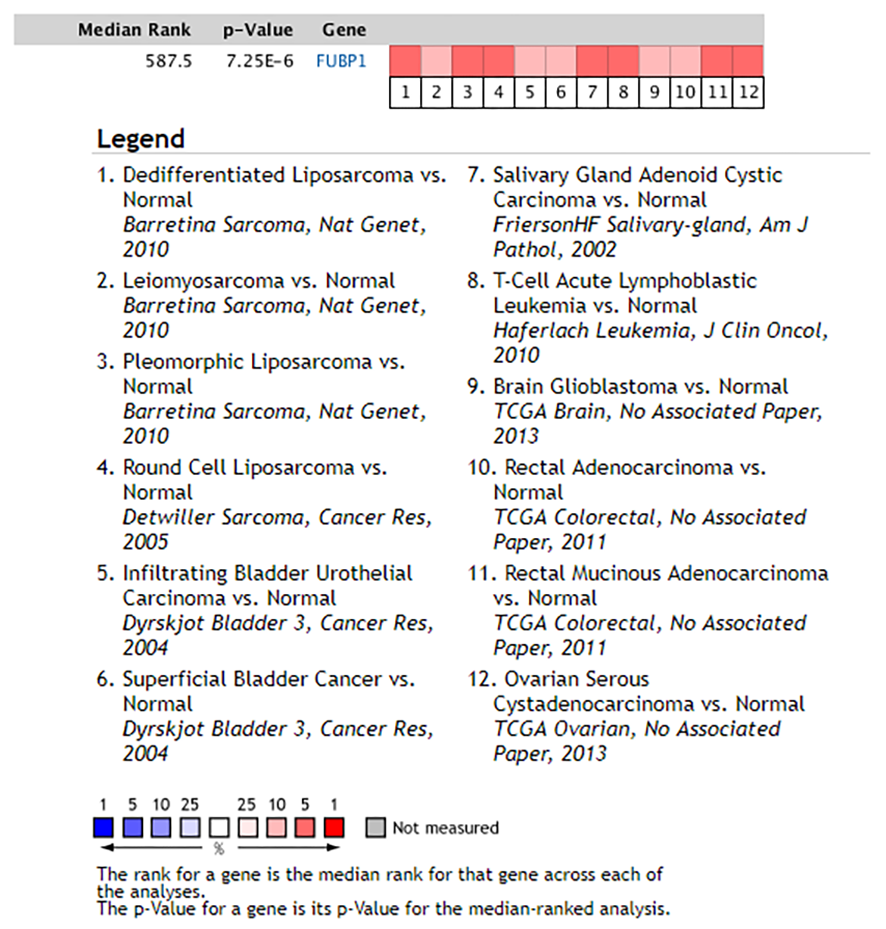

Supplement: Supplementary file 16 [file Image5.TIF]

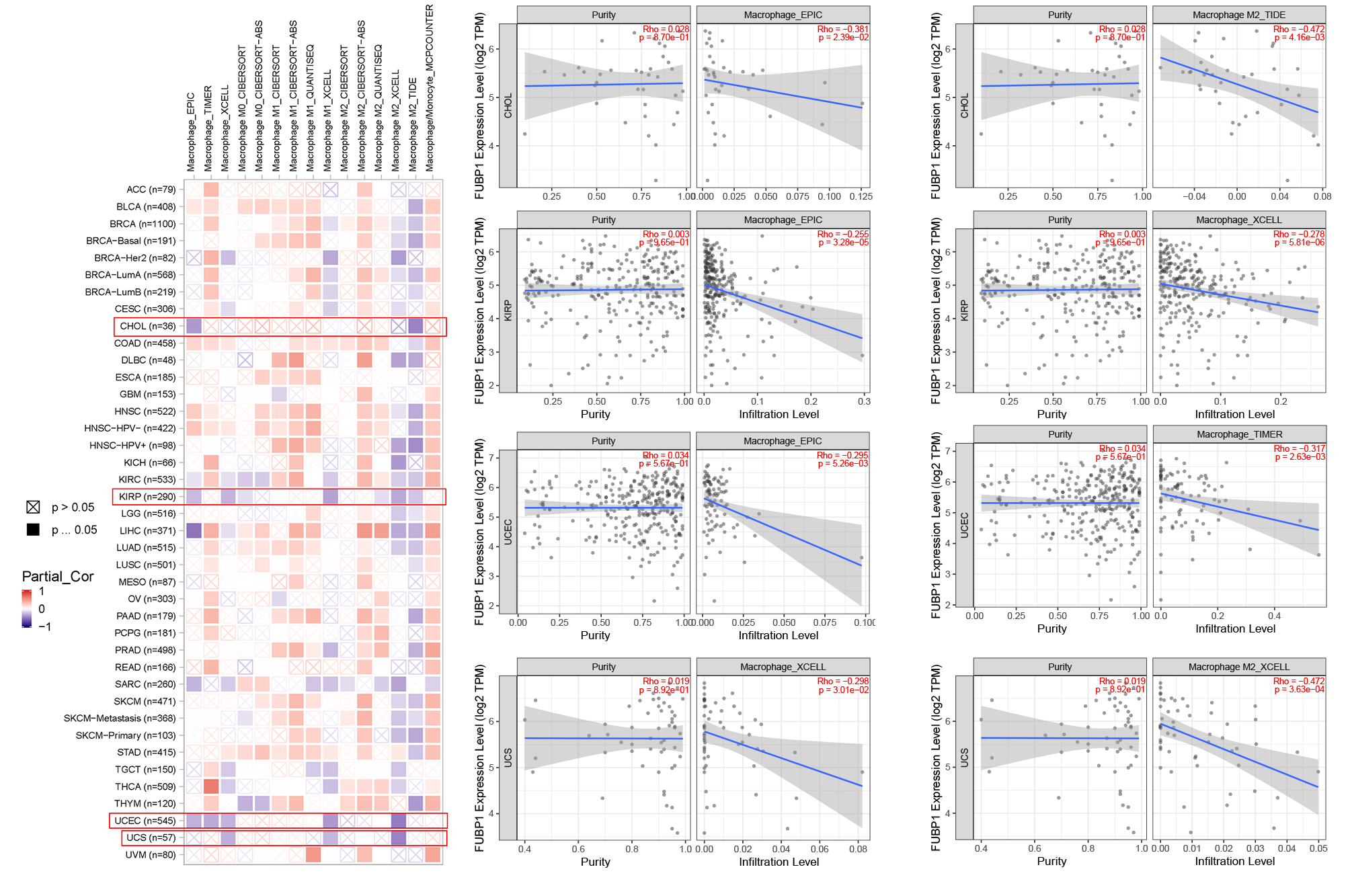

Supplement: Supplementary file 17 [file Image12.TIF]
