# Supplementary material for: Pan-Cancer Transcriptome and Immune Infiltration Analyses Reveal the Oncogenic Role of Far Upstream Element-Binding Protein 1 (FUBP1)
Source: Front Mol Biosci. 2022 Feb 22;9:794715. doi: 10.3389/fmolb.2022.794715 (PMC8902172; doi:10.3389/fmolb.2022.794715)
Supplement: Supplementary file 3 [file Table1.DOCX]

| **Accession Number** | **Website Link** |
| --- | --- |
| ARV77948 | <https://www.ncbi.nlm.nih.gov/protein/ARV77948.1> |
| XP_029083935 | https://www.ncbi.nlm.nih.gov/protein/XP_029083935.1 |
| XP_032969090 | https://www.ncbi.nlm.nih.gov/protein/XP_032969090.1 |
| XP_042106022 | https://www.ncbi.nlm.nih.gov/protein/XP_042106022.1 |
| XP_040088446 | https://www.ncbi.nlm.nih.gov/protein/XP_040088446.1 |
| XP_035307141 | https://www.ncbi.nlm.nih.gov/protein/XP_035307141.1 |
| XP_035882224 | https://www.ncbi.nlm.nih.gov/protein/XP_035882224.1 |
| XP_036303281 | https://www.ncbi.nlm.nih.gov/protein/XP_036303281.1 |
| XP_036205794 | https://www.ncbi.nlm.nih.gov/protein/XP_036205794.1 |
| XP_038267857 | https://www.ncbi.nlm.nih.gov/protein/XP_038267857.1 |
| XP_030349804 | https://www.ncbi.nlm.nih.gov/protein/XP_030349804.1 |
| XP_033261833 | https://www.ncbi.nlm.nih.gov/protein/XP_033261833.1 |
| NP_001032742 | https://www.ncbi.nlm.nih.gov/protein/NP_001032742.1 |
| AAH14763 | https://www.ncbi.nlm.nih.gov/protein/ AAH14763.1 |
| XP_036091356 | https://www.ncbi.nlm.nih.gov/protein/XP_036091356.1 |
| XP_036111158 | https://www.ncbi.nlm.nih.gov/protein/XP_036111158.1 |
| XP_035573744 | https://www.ncbi.nlm.nih.gov/protein/XP_035573744.1 |
| XP_038526269 | https://www.ncbi.nlm.nih.gov/protein/XP_038526269.1 |
| GSE131013 | https://www.ncbi.nlm.nih.gov/geo/query/acc.cgi?acc=GSE131013 |

**Accession numbers and website links of GEO data**
