# Supplementary material for: Pan-Cancer Transcriptome and Immune Infiltration Analyses Reveal the Oncogenic Role of Far Upstream Element-Binding Protein 1 (FUBP1)
Source: Front Mol Biosci. 2022 Feb 22;9:794715. doi: 10.3389/fmolb.2022.794715 (PMC8902172; doi:10.3389/fmolb.2022.794715)
Supplement: Supplementary file 14 [file DataSheet1.PDF]

Supplementary Material

Supplementary Tables

Table S1. Subgroup analysis on the correlation of *FUBP1* and the prognosis of breast cancers.

Factor	Subgroup	Sample size	OS		RFS		DMFS	
			HR	*P*	HR	*P*	HR	*P*
**ER status**	ER positive	3499	1.14	0.4	1	0.95	1.18	0.23
	ER negative	2168	0.94	0.7	0.97	0.76	0.87	0.3
**TP53 status**	mutated	272	1.47	0.27	1.48	0.11	1.97	0.082
	Wild type	388	0.97	0.92	1.42	0.11	1.59	0.22
**PR status**	PR positive	1559	0.92	0.82	1.08	0.6	1.18	0.47
	PR negative	1989	1.33	0.26	1.1	0.41	0.99	0.97
**HER2 status**	HER2 positive	1273	1.34	0.11	0.86	0.19	0.98	0.88
	HER2 negative	6262	0.89	0.29	0.9	0.081	1.01	0.94
**Grade**	Grade 1	576	1.04	0.94	1.15	0.6	1.9	0.12
	Grade 2	1795	0.92	0.69	0.91	0.4	0.92	0.57
	Grade 3	2058	1.12	0.45	1.1	0.3	1.16	0.27
**Subtype - StGallen**	Basal	1494	0.69	0.059	0.97	0.77	0.8	0.17
	Luminal A	3511	0.82	0.21	0.81	0.097	0.93	0.6
	Luminal B	2015	1.42	0.05	0.99	0.93	1.37	**0.028**
	HER2+	515	1.21	0.52	0.72	0.068	0.73	0.21
**Lymph node status**	Lymph node positive	2153	1.12	0.52	1.07	0.45	1.09	0.5
	Lymph node negative	2829	0.94	0.74	1.07	0.43	1.08	0.52
**Pietenpol subtype**	Basal-like 1	418	0.94	0.87	1.18	0.44	1.01	0.96
	Basal-like 2	165	1.35	0.55	1.83	0.053	1.2	0.63
	immunomodulatory	462	2.21	0.058	1.55	0.061	1.27	0.39
	Mesenchymal	382	0.52	0.052	1.07	0.75	0.57	0.065
	Mesenchymal stem-like	201	1.55	0.41	1.68	0.15	1.13	0.82
	Luminal androgen receptor	413	1.23	0.5	0.62	**0.014**	0.86	0.6

OS, overall survival; RFS, relapse free survival; DMFS, distant metastasis free survival; HR, hazard ratio; ER, Estrogen receptor; PR, Progesterone receptor; NA, not available; *P* values $<$ 0.05 is considered statistically significant.

Table S2. Subgroup analysis on the correlation of *FUBP1* and the prognosis of lung cancers.

Factor	Subgroup	Sample size	OS		FP		PPS	
			HR	*P*	HR	HR	*P*	HR
**Histology**	adenocarcinoma	866	2.18	**1.8e-10**	1.37	**0.045**	1.3	0.26
	squamous cell carcinoma	675	1.03	0.8	0.68	0.14	1.12	0.83
**gender**	female	818	1.72	**4.6e-06**	1.64	**0.00068**	1.77	**0.0027**
	male	1387	1.25	**0.0047**	1.33	**0.031**	1.06	0.76
**smoking history**	exclude those never smoked	970	1.45	**0.00046**	1.34	**0.018**	1.2	0.23
	only those never smoked	247	3.65	**1.9e-05**	1.93	**0.007**	1.65	0.11
**stage**	stage I	652	2.57	**1.8e-11**	1.07	0.77	1.85	**0.042**
	stage II	320	1.7	**0.004**	0.79	0.36	1.65	0.13
	stage III	70	0.93	0.81	NA	NA	NA	NA
**grade**	grade I	202	0.99	0.97	0.96	0.84	0.97	0.92
	grade II	310	1.3	0.097	1.03	0.89	1.6	0.056
	grade III	77	1.54	0.2	1.19	0.67	1.13	0.81
**AJCC stage t**	t1	475	1.16	0.3	1.43	0.16	1.37	0.32
	t2	686	1	0.98	1.19	0.25	0.96	0.82
	t3	99	0.92	0.74	1.11	0.83	NA	NA
	t4	48	1.45	0.26	NA	NA	NA	NA
**AJCC stage n**	n0	863	1.12	0.28	1.2	0.27	1.14	0.51
	n1	296	1.1	0.54	1.43	0.12	1.03	0.92
	n2	113	1.03	0.87	0.68	0.27	0.58	0.13
**AJCC stage m**	m0	818	1.1	0.38	0.96	0.87	0.76	0.39
**surgery**	only surgical margins negative	730	1.82	**4.1e-07**	1.89	**6.8e-07**	1.55	**0.0038**
**radiotherapy**	no	276	1.06	0.76	1.04	0.85	1.14	0.56
	yes	73	1.02	0.95	0.92	0.77	0.97	0.91
**chemotherapy**	no	317	1.27	0.16	0.88	0.51	1.17	0.52
	yes	178	1.28	0.24	1.21	0.36	1.11	0.65

HR, hazard ratio; OS, overall survival; FP, first progression; PPS, post progression survival; NA, not available data; AJCC, American Joint Committee on Cancer; *P* values＜0.05 is considered statistically significant.

Table S3. Subgroup analysis on the correlation of *FUBP1* and the prognosis of ovarian cancers.

Factor	Subgroup	Sample size	OS		PFS		PPS	
			HR	*p*	HR	*p*	HR	*p*
**Histology**	Endometrioid	62	0.23	0.084	1.92	0.16	NA	NA
	Serous	1232	1.08	0.34	1.36	**4.8e-05**	1.1	0.3
**Stage**	Stage 1	107	1.89	0.33	2.87	**0.048**	NA	NA
	Stage 2	72	1.99	0.22	2.48	**0.011**	1.99	0.27
	Stage 3	1079	0.91	0.23	1.3	**0.0032**	1.14	0.21
	Stage 4	189	0.64	**0.049**	1.66	**0.011**	0.68	0.14
**Grade**	Grade 1	56	0.49	0.2	1.46	0.53	NA	NA
	Grade 2	325	0.8	0.16	1.29	0.094	1.34	0.19
	Grade 3	1024	0.87	0.1	1.25	**0.0086**	0.9	0.35
	Grade 4	21	1.91	0.18	NA	NA	NA	NA
**TP53 mutation**	Mutated	516	1.31	**0.027**	1.69	**1.4e-05**	1.14	0.33
	Wild type	102	0.6	0.096	1.93	**0.024**	1.7	0.095
**Debulk**	optimal	802	0.78	**0.017**	1.2	0.082	0.75	0.065
	suboptimal	536	0.91	0.37	1.22	0.071	1.2	0.18
**Chemotherapy**	Contains platin	1438	0.91	0.21	1.21	**0.013**	0.92	0.34
	Contains Taxol	821	0.88	0.2	1.2	**0.034**	0.88	0.22
	Contains Taxol+platin	804	0.87	0.17	1.23	**0.031**	0.87	0.21
	Contains Avastin	50	0.53	0.22	0.48	**0.031**	0.52	0.2
	Contains Docetaxel	108	0.48	**0.022**	0.73	0.27	0.53	0.053
	Contains Gemcitabine	135	0.86	0.43	0.84	0.39	1.24	0.37
	Contains Paclitaxel	248	0.66	0.13	1.29	0.16	0.56	0.077
	Contains Topotecan	119	0.88	0.52	1.16	0.52	0.83	0.36

HR, hazard ratio; OS, overall survival; PFS, progress free survival; PPS, post progression survival; TP53, Tumor Protein P53; NA, not available data; *P* values＜0.05 is considered statistically significant.

Table S4. Subgroup analysis on the correlation of *FUBP1* and the prognosis of liver cancers.

Factor	Sub group	Sample size	OS		PFS		RFS		DSS	
			HR	*P*	HR	*P*	HR	*P*	HR	*P*
**Stage**	Stage 1	171	2.36	**0.0067**	0.62	0.094	0.65	0.13	0.49	0.13
	Stage 2	86	0.58	0.19	1.86	**0.036**	1.51	0.22	4.59	0.11
	Stage 3	85	3.15	**0.0003**	1.71	0.087	1.87	0.054	4.09	**0.0001**
**Grade**	Grade 1	55	8.46	**2.6e-05**	1.6	0.23	0.44	0.12	9.34	**0.0021**
	Grade 2	177	1.59	0.12	1.58	0.055	0.71	0.18	1.95	0.14
	Grade 3	122	2.35	**0.0042**	1.85	**0.022**	1.99	**0.011**	2.8	**0.0057**
**AJCC_T**	T1	181	2.28	**0.0059**	0.63	0.087	0.63	0.13	1.61	0.29
	T2	94	1.74	0.26	1.82	**0.029**	1.5	0.22	5.71	0.056
	T3	80	3.34	**0.00014**	1.5	0.16	1.73	0.092	3.11	**0.0045**
**Gender**	Female	121	1.76	0.062	1.38	0.27	0.75	0.37	1.93	0.13
	Male	250	2.92	**1.6e-06**	1.89	**6e-04**	2.06	**0.00044**	2.96	**0.00015**
**Vascular invasion**	None	205	2.93	**5.4e-05**	1.49	0.11	1.53	0.095	2.3	**0.03**
	micro	93	0.56	0.15	1.63	0.095	0.51	**0.034**	0.31	0.064
**Race**	White	184	1.93	**0.014**	1.6	**0.019**	1.82	**0.0092**	2.85	**0.0023**
	Asian	158	4.29	**2.3e-07**	2.31	**0.0012**	2.02	**0.011**	3.35	**0.0018**
**Sorafenib treatment**	treated	30	0.59	0.34	1.59	0.24	2.41	0.056	0.59	0.34
**Alcohol consum ption**	Yes	117	2.56	**0.005**	2.16	**0.0041**	2.93	**0.00037**	2.76	**0.0076**
	none	205	2	**0.0042**	1.61	**0.033**	0.7	0.13	2.21	**0.017**
**Hepatitis virus**	Yes	153	2.95	**0.00061**	2.07	**0.0033**	1.58	0.085	2.79	**0.01**
	none	169	1.97	**0.0065**	1.59	**0.049**	1.61	0.083	2.47	**0.013**

OS, overall survival; PFS, progress free survival; RFS, relapse free survival; DSS, disease specific survival; HR, hazard ratio; AJCC，American Joint Committee on Cancer; *P* values＜0.05 is considered statistically significant.

Table S5. Specific information of CPTAC-identified phosphorylation sites of FUBP1 via the database of PhosphoNET.

Site	Sequence	Experimentally confirmed[#]	Hydrophobicity	p-site similarity score	Maximum kinase specificity	Sum kinase specificity score	Conservation score
**S55**	TSLN**S**NDYG	NA	-1.100	-60.5	434	11644	29.7
**S99**	QQSR**S**VMTE	NA	-1.753	-63.7	289	12751	24.2
**S120**	GEQI**S**RIQQ	NA	-1.487	-57.6	325	12306	31.8
**S140**	IAPD**S**GGLP	NA	-0.767	-56.0	335	13802	40.6
**T153**	MLTG**T**PESV	15302935	-0.367	-55.7	350	10584	34.6
**S630**	YAQT**S**PQM	15302935	-0.993	-62.8	494	20233	27.3

[#]The PMID of the publication; NA, not available.
